# Microenvironment‐Responsive Drug Delivery Systems for Ocular Surface Diseases: Mechanisms, Applications, and Translational Challenges

**DOI:** 10.1002/smsc.70354

**Published:** 2026-08-02

**Authors:** Jiamin Liu, Liangbo Chen, Yao Fu, Siyi Zhang

**Affiliations:** ^1^ Department of Ophthalmology Ninth People's Hospital Shanghai Jiao Tong University School of Medicine Shanghai China; ^2^ Shanghai Key Laboratory of Orbital Diseases and Ocular Oncology Shanghai China

**Keywords:** characterization techniques, dry eye disease, infectious keratitis, microenvironment‐responsive drug delivery, translational barriers

## Abstract

Vision impairment not only compromises patients’ quality of life but also imposes a substantial societal and economic burden. Conventional ophthalmic formulation is hindered by ocular physiological barriers, leading to low bioavailability and necessitating frequent administration. To overcome these limitations, novel microenvironment‐responsive drug delivery systems (MR‐DDSs), have emerged as a highly promising strategy. This review aims to systematically summarize recent advances in MR‐DDSs for treating ocular diseases, providing insights for clinicians and researchers. Firstly, we elucidated the characteristics of the ocular microenvironment under normal and pathological conditions, including factors such as temperature, pH, and the levels of ions, reactive oxygen species (ROS), and enzymes. Subsequently, we introduced the categories, properties, and molecular mechanisms of stimulus‐responsive polymers, followed by a summary of preparation methods, characterization techniques and performance metrics for major systems. Then, we summarized the application and therapeutic efficacy of MR‐DDSs in managing a range of common ocular surface diseases, including dry eye disease, infectious keratitis, and corneal neovascularization. Finally, we critically analyzed the obstacles to clinical translation and propose promising avenues for future research. This review offers a comprehensive framework bridging material design, performance evaluation, and clinical translation, providing actionable insights for both clinicians and researchers in the field.

## Graphical abstract

Microenvironment‐responsive drug delivery systems overcome ocular physiological barriers by responding to temperature, pH, ions, reactive oxygen species, and enzymes. This review bridges material design, performance evaluation, and clinical translation, offering actionable insights for treating dry eye disease, infectious keratitis, and corneal neovascularization. Key translational challenges and future research directions are critically analyzed.

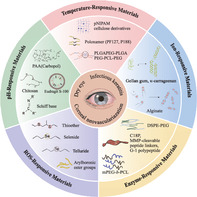

**Abbreviations**
¹H NMR: Proton nuclear magnetic resonance spectroscopy
ACPA: Allylaminocarbonylphenylboronic acid
ADA: Adalimumab
AGV: Aqueous glaucoma valve
AK: Acanthamoeba keratitis
ALP: Alkaline phosphatase
AMD: Age‐related mascular degeneration
Amp: Ampicillin
AZA: Acetazolamide
BMP4: Bone morphogenetic protein 4
BSA: Bovine serum albumin
CAP: Cellulose acetate phthalate
CAT: Catalase
CFU: Colony‐forming units
CIP: Ciprofloxacin
CLs: Contact lenses
CNF: Cellulose nanofiber
CNV: Corneal neovascularization
COL I: Type I collagen
CS: Chitosan
CsA: Cyclosporine A
DD: Degree of deacetylation
DDSs: Drug delivery systems
DED: Dry eye disease
Dex: Dexamethasone sodium phosphate
DLS: Dynamic light scattering
DR: Diabetic retinopathy
DSPE: 1,2‐Distearoyl‐sn‐glycero‐3‐phosphoethanolamine
EB: 4‐Carboxyphenylboronic acid pinacol ester
ECM: Extracellular matrix
EGCG: Epigallocatechin gallate
FA: Folic acid
Feno: Fenofibrate
FK: Fungal keratitis
FTIR: Fourier transform infrared
GMS: Gel/microsphere
GP: B‐glycerolphosphate
GPX: Glutathione peroxidase
HAMA: Methacrylated hyaluronic acid
HCEC: Human corneal epithelial cells
HIF: Hypoxia‐inducible factor
HO: Heme oxygenase
HPMC: Hydroxypropyl methylcellulose
HTCC: N‐(2‐Hydroxy‐3‐trimethylammonium) propyl chitosan chloride
IK: Infectious keratitis
IOP: Intraocular pressure
LCST: Lower critical solution temperature
LPS: Lipopolysaccharide
MEMA: 2‐(Methylthio)ethyl methacrylate
MMPs: Matrix metalloproteinases
MN: Microneedle
MR: Microenvironment‐responsive
MRSA: Methicillin‐resistant *Staphylococcus aureus*
NLC: Nanostructured lipid carriers
NMs: Nanomicelles
NPs: Nanoparticles
OCMC: Oxidized carboxymethyl chitosan
OSA: Oxidized sodium alginate
P188: Poloxamer 188
P407/F127: Poloxamer 407
PAA: Poly (acrylic acid)
PCL: Poly(ε‐caprolactone)
PDI: Polydispersity index
PDMS: Polydimethylsiloxane mold
PEG: Poly (ethylene glycol)
PEI: Polyethylenimine
PEL: E‐Polylysine
PEO: Polyethylene oxide
PI: Propamidine isethionate
PLA: Polylactic acid
PLGA: Hydrophobic poly (lactic acid‐co‐glycolic acid)
pNIPAAm: Poly (N‐isopropylacrylamide)
POSS: Polyhedral oligomeric silsesquioxane
PPG: Polypropylene glycol
PPO: Polypropylene oxide
PPP: PLGA‐PEG‐PLGA
PPS: Poly (propylene sulfide)
PS: Particle size
ROS: Reactive oxygen species
RSV: Resveratrol
SEM: Scanning electron microscopy
SI‐RAFT: Surface‐initiated reversible addition–fragmentation chain transfer
SOD: Superoxide dismutase
SRPs: Stimulus‐responsive polymers
SSDE: Sjögren's syndrome‐related dry eye
TBC: Tributyl citrate
TEM: Transmission electron microscopy
TK: thioketal
UCST: Upper critical solution temperature
VEGF: Vascular endothelial growth factor
VOR: Voriconazole
WHO: World Health Organization

## 1 Introduction

Visual impairment represents a profound public health challenge globally. It was estimated that 216.6 million people lived with moderate‐to‐severe vision impairment, accounting for 2.95% of the world's population, among whom 36 million were blind [1]. Leading ocular diseases such as glaucoma, age‐related macular degeneration (AMD), and infectious keratitis (IK), severely diminish the quality of life for affected individuals and impose a substantial socioeconomic burden [2, 3]. Consequently, the World Health Organization (WHO) prioritizes eye health as an essential global issue to reduce preventable blindness and vision impairment [4].

**FIGURE 1:**
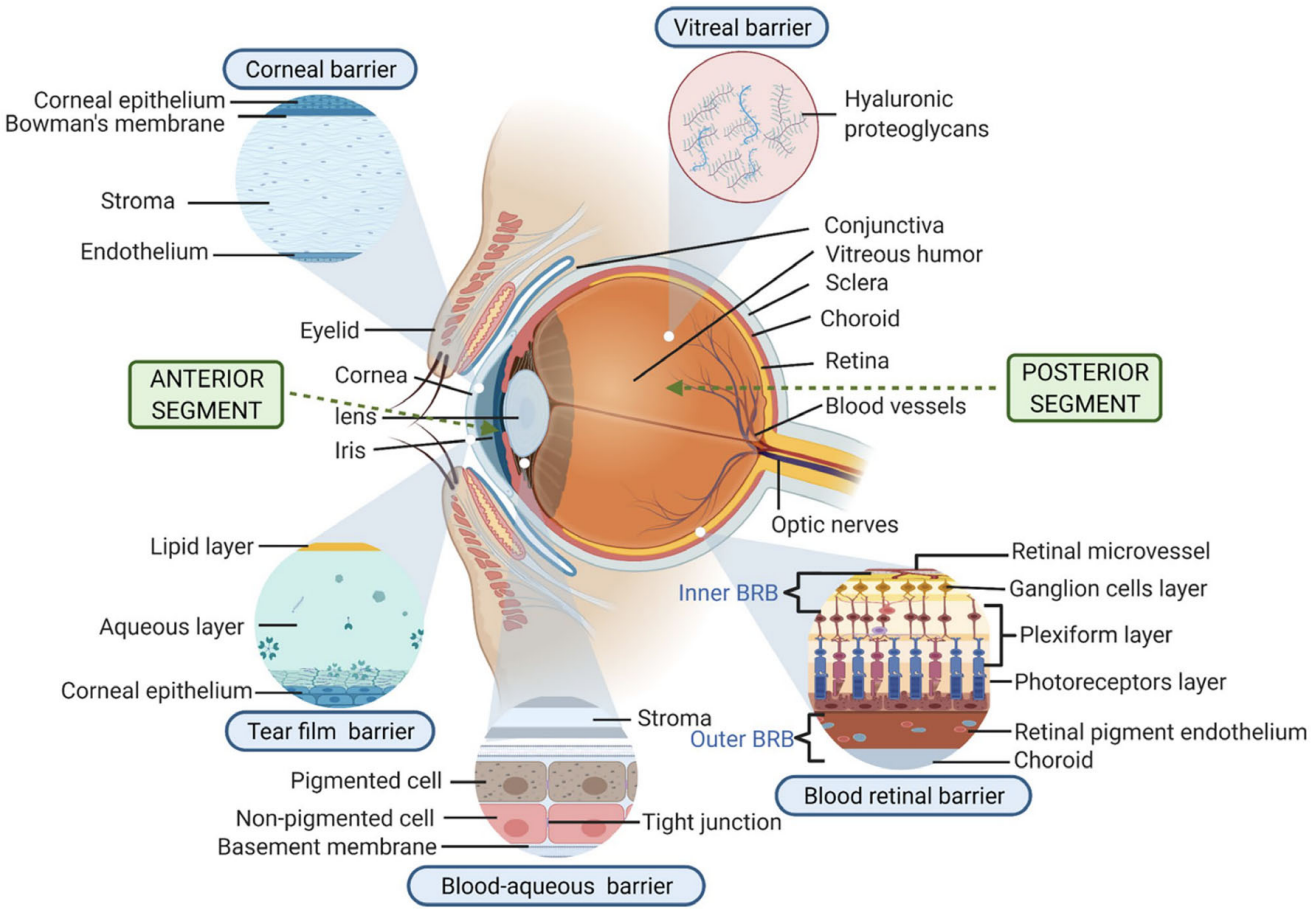
Ocular physiological barriers (tear film, cornea, vitreous barrier, blood‐aqueous barrier, and blood‐retina barrier) that limit drug penetration and distribution. Reproduced under the terms of the Creative Commons Attribution 4.0 International License, ref. [11] Copyright 2025, Springer.

The primary treatment for these diseases relies on conventional topical formulations, such as eye drops, ointments, and gels. However, the therapeutic efficacy is fundamentally limited by the eye's intrinsic anatomical and physiological barriers [5, 8]. These include static structures such as the tear film, cornea, vitreous barrier, blood‐aqueous barrier, and blood‐retina barrier, which collectively impede drug penetration and distribution [9, 11] (Figure 1). Furthermore, rapid tear turnover and nasolacrimal drainage significantly reduce drug residence time, resulting in extremely low bioavailability. It is estimated that typically less than 5% of the administered dose reaching the target tissues [10, 12]. This inefficient delivery necessitates frequent and high‐dose regimens, which in turn increases the risk of local side effects, ocular toxicity, and systemic absorption [9].

Overcome these limitations requires novel and efficient drug delivery systems (DDSs). Among emerging strategies, microenvironment‐responsive DDSs (MR‐DDSs), integrating stimulus‐responsive polymers (SRPs) with diverse drug delivery platforms (e.g., hydrogel, nanomaterials, or microneedles), show considerable promise [13, 17]. In contrast to the passive release of conventional formulations, SRPs can sense specific pathological signals in the ocular microenvironment and respond with physical or chemical transformations to enable on‐demand drug release at the target site [18, 19]. For instance, inflammation exhibit elevated reactive oxygen species (ROS) and overexpressed enzymes such as matrix metalloproteinases (MMPs) [20, 22]. By harnessing these pathological triggers, SRPs based drug carriers can be engineered to activate at the disease site, enhancing therapeutic efficacy while minimizing off‐target effects. Additionally, the controlled release profile of novel DDSs enhances bioavailability and patients’ comfort [23].

This review summarized the advances over the past decade in novel DDSs based on SRPs for treating ocular diseases. First, we outlined the characteristics of the ocular microenvironment under normal and pathological conditions, including temperature, pH, ions, ROS, and enzymes. Then, we categorized the types, properties, and response mechanisms of current SRPs. Subsequently, we focused on the application and therapeutic efficacy of MR‐DDSs in managing common ocular surface disorders, including dry eye disease (DED), infectious keratitis (IK) and corneal neovascularization (CNV). Finally, we discussed the current challenges and future directions for the clinical translation of MR‐DDSs. This review was the first to systematically integrate SRPs properties, performance evaluation, application and clinical translation barriers within a single framework, thereby offering a roadmap for translational research in this field.

## 2 The Ocular Microenvironment in Physiological and Pathological States

The ocular system maintains a precise and stable internal microenvironment, which is essential for its function. Under disease conditions, this homeostasis is disrupted, leading to distinct pathological alterations. A summary comparison of the ocular microenvironment under physiological and pathological conditions is provided in Table 1. These disease‐specific changes such as shifts in pH, enzyme activity, or ROS levels represent exploitable targets for smart DDSs. Understanding these pathological changes serves as the cornerstone for designing targeted and efficient MR‐DDSs.

**TABLE 1:** Major physicochemical factors of the ocular microenvironment and the alterations under pathological conditions.

Factor	Physiological state	Pathological alterations	Associated diseases
Temperature	34.79 ± 0.68 °C at corneal surface and 36.08 ± 0.62 °C at the inner canthus	Elevation due to inflammation	IK [24]
		Reduction due to excessive tear evaporation or reduced blood perfusion	DED, glaucoma, AMD, DR [25, 27]
pH	Neutral pH (approximately 7.0), ranging from 6.5 to 7.6	Shift toward acidity under inflammation, infection, and ischemia	IK, glaucoma, DR [28, 29]
Ion	Ions like Na^+^, Cl^−^, HCO_3_^−^, Ca^2+^, K^+^, and Mg^2+^ maintain homeostatic osmolarity (~300 mOsm/L)	Hyperosmolarity (elevated ionic concentrations)	DED, diabetic senile cataract patients [30, 31]
ROS	Low basal levels maintained by redox homeostasis	Oxidative stress (ROS overproduction)	DED, pterygium, cataract, uveitis, glaucoma, DR, and AMD [32, 35]
Enzyme	Low levels of MMPs, ALP, hyaluronidase and lysozyme	Elevated levels of MMPs	DED, CNV, IK, and glaucoma [36, 39]
		Elevated phosphatase, phospholipase, esterase, lipase, and hyaluronidase	IK [40, 42]
		Elevated ALP	Uveitis, corneal injury [43, 44]

### 2.1 Temperature

The ocular temperature is tightly regulated under normal conditions, with the corneal surface averaging 34.79 ± 0.68 °C and the inner canthus at 36.08 ± 0.62 °C, while intraocular temperature remains stable near core body temperature [45]. Pathological states associated with reduced ocular blood perfusion can lead to a decrease in ocular surface temperature, such as glaucoma and age‐related macular degeneration (AMD) [25, 26]. For instance, Sodi et al. found the corneal central temperature of AMD patients was 34.11 ± 0.54 °C, significantly lower than the controls (34.64 ± 0.84 °C). Abreau et al. further demonstrated that DED also correlates with reduced temperature, likely due to impaired tear film stability. In their study, relative to normal eyes (35.11 ± 0.64 °C), evaporative dry eyes (EDE) had lower central corneal temperature (34.16 ± 1.12 °C) [27]. Conversely, inflammation often induces localized hyperthermia due to increased blood flow and metabolic activity. Klamann et al. observed significantly elevated temperatures in patients with bacterial corneal ulcers (35.6 °C ± 0.9) than controls (34.8 °C ± 0.8) [24].

### 2.2 pH

The healthy ocular microenvironment maintains a neutral pH (approximately 7.0), with tears ranging from 6.5 to 7.6 and intraocular fluids residing within a similar range [46, 47]. Pathological conditions like inflammation, infection, and ischemia can lead to the accumulation of acidic metabolites because of enhanced anaerobic glycolysis, which then result in acidotic extracellular pH levels ranging from pH 5.5–7.0 [48]. Lu et al. reported aqueous humor acidosis in patients with acute primary angle closure, where a lower pH correlated with higher intraocular pressure and/or longer duration of elevated intraocular pressure (IOP). Similarly, retina ischemia and hypoxia in DR promote an accumulation of acidic metabolites, exacerbating local acidosis and contributing to further retinal cell damage [28].

### 2.3 Ion

In the healthy eyes, the tear film, aqueous humor, and vitreous each maintain a distinct ionic equilibrium, involving electrolytes such as Na^+^, Cl^−^, HCO_3_^−^, Ca^2+^, K^+^, and Mg^2+^ to preserve corneal epithelial integrity, lens transparency, and retinal function. These ions also play essential roles in regulating osmotic pressure, pH balance, and cellular homeostasis across ocular tissues [49]. Under pathological conditions, however, this delicate ionic balance is frequently disrupted. In DED, excessive tear evaporation elevates tear film osmolarity (>308 mOsm/L) and increases ionic concentrations, leading to epithelial damage and aggravated inflammation [30]. In diabetic senile cataract patients, the serum sodium levels was 144.16 ± 2.25 meq/L, which was notably higher than the 140.26 ± 3.60 meq/L observed in the non‐diabetic control group [31].

### 2.4 ROS

ROS, primarily including hydroxyl radicals (·OH), superoxide anions (O_2_^·^^−^O_2_^·−^), singlet oxygen (^1^O2), and hydrogen peroxide (H_2_O_2_), are natural byproducts of cellular metabolism [50]. Under physiological conditions, ROS levels are tightly regulated by an endogenous antioxidant defense system, comprising enzymes such as superoxide dismutase (SOD), catalase (CAT), heme oxygenase (HO), and glutathione peroxidase (GPX), which protects cellular macromolecules (e.g., DNA, proteins, and lipids) from oxidative damage [51, 52]. However, factors like aging, hyperglycemia, and UV exposure can disrupt this balance, leading to ROS overproduction [53]. Moreover, ROS activates inflammatory pathways (MAPKs, NF‐κB), leading to the upregulation of cytokines like TNF‐α and IL‐1β, which in turn propagate a vicious cycle of oxidative stress and inflammation [54]. This sustained oxidative stress serve as a critical pathogenic driver in multiple ocular diseases, including DED, pterygium, cataract, uveitis, glaucoma, DR, and AMD [32, 35]. For instance, the level of malondialdehyde (MDA), as oxidative stress marker, in the cornea of DED patients was 30.9 ± 21.1 pmol/mL, significantly higher than that in normal controls, which was 12.1 ± 3.16 pmol/mL [55]. The elevated ROS in diseases provides a specific trigger for developing ROS‐responsive DDSs [56].

### 2.5 Enzymes

Under physiological conditions, ocular enzymes such as matrix metalloproteinases (MMPs), alkaline phosphatase (ALP), hyaluronidase and lysozyme maintain a homeostatic balance, mainly involved in metabolism and defense. However, abnormal overexpression of specific enzymes is implicated in multiple ocular pathologies. Studies have reported elevated levels of MMP‐9 expression in patients with ocular surface injuries, DED, and CNV [36, 37]. For example, Marcela et al. found that tear MMP‐9 values were 1395.0 ± 453.2 ng/ml in lagophthalmic eyes, significantly higher than in healthy eyes (278.9 ± 79.0 ng/ml) [57]. In bacterial keratitis, CXCL16 can exacerbates disease by increasing corneal bacterial load, promoting neutrophil infiltration, and amplifying local ROS and MMP production [38]. Further host damage is mediated through the bacterial secretion of destructive enzymes, including phosphatase, phospholipase, esterase, lipase, and hyaluronidase [40, 42]. Li et al. investigated MMPs expression during the progress o*f Fusarium solani* (*F. solani*) keratitis in a rat model and found that MMP‐3 expression in the early stage was 66.3 times higher in infected corneas compared with normal corneas. In the mid‐stage of the infection, MMP‐8, ‐9, and ‐13 expressions were upregulated, with infected‐to‐normal ratios of 4.03, 39.86, and 5.94, respectively. While in the late stage, MMP‐2 and ‐7 expressions increased with the infected‐to‐normal ratios of 5.94 and 16.22, respectively [58]. Furthermore, Hu et al. found that the expression of ALP in the tear fluid of uveitis patients (586.43 ± 470.86 U/L) was much higher than that of uveitis‐free people (165.25 ± 128.58 U/L) [43]. The elevation of these specific enzymes provides highly selective targets for the design of enzyme‐responsive DDSs.

## 3 Classifications and Mechanisms of Stimulus‐Responsive Materials

Stimulus‐responsive materials can be engineered into various formulations, such as hydrogels, nanoparticles (NPs), micelles, microneedles (MNs), contact lenses (CLs), and implants. These materials are generally classified by their triggering signals (Table 2). According to the ocular pathological features, five major classes are discussed in this section: temperature‐responsive, pH‐responsive, ion‐responsive, ROS‐responsive, and enzyme‐responsive polymers. The chemical structures and response mechanisms of each class are described. The subsequent sections will address how these polymers are engineered into various delivery forms and characterized.

**TABLE 2:** Summary of representative stimulus‐responsive polymers.

Stimulus	Polymers	Advantages	Limitations	References
Temperature	Poloxamers	Amphiphilicity, good biocompatibility, easy sterilization	Weak mechanical properties	[59, 60]
	pNIPAAm	Excellent thermos‐sensitive sol–gel transition behavior	limited biodegradability, weak mechanical properties	
	PLGA‐PEG‐PLGA	Biodegradable, tunable gelation/release kinetics, good sol stability	Complex synthesis, acidic degradation products may cause inflammation	[61, 62]
	PEG‐PCL‐PEG	Combines biocompatibility with long‐term, sustained release capability	Slow degradation of PCL, the formed hydrogel is opaque	[63]
pH	Carbopol	Rapid, reversible pH‐swelling, excellent mucoadhesion ability	High viscosity at low pH (irritation risk), requires neutralization	[64]
	Chitosan	Biocompatibility, nontoxicity, mucoadhesive, antibacterial and antifungal properties	Gelation/properties highly pH‐dependent, batch‐to‐batch variability	[23, 65]
	PEI	Nonviral gene delivery vector, superior pH buffering capacity	High cytotoxicity, poor stability	[66]
	Eudragit S100	Precisely dissolves and releases drug at elevated pH	Poor hydrophilicity, affects ocular comfort	[67]
	Schiff base	Dynamic covalent bond, can quickly hydrolyze under weakly acidic condition	Hydrolysis instability in strongly acidic media	[68]
Ion	Gellan gum	Rapid ion‐triggered gelation, high transparency, thermo‐reversible	Brittle gel, batch‐to‐batch variability	[64, 69]
	κ‐Carrageenan	Forms strong, elastic gels with potassium ions	Gel syneresis over time, leading to shrinkage and drug leakage	[70]
	Alginate	Biocompatibility, biodegradability, nontoxicity, and nonimmunogenicity	Brittle gel, batch‐to‐batch variations	[71]
ROS	Thioketal‐based	Highly specific cleavage by ·OH, not by H_2_O_2_	Hydrolysis risk in acidic microenvironments	[72, 73]
	PPS	Excellent ROS scavenging, gradual hydrophobic‐to‐hydrophilic switch	Slow response kinetics, may lack sharp drug release	[74, 75]
	Se‐PEG‐PPG	Higher oxidative sensitivity and lower redox potential	Potential selenium‐related toxicity at high doses	[76]
	Arylboronic Ester‐based	Fast and sensitive response to H_2_O_2_	Susceptible to hydrolysis, compromising stability	[77]
Enzyme	C18P	MMP9‐responsive release	Requires co‐assembly for drug delivery	[42]
	MMP‐cleavable peptide linkers (cL)	Specifically cleaved by MMPs	Limited drug loading capacity, prone to proteolysis	[78]
	PCL	Sensitive to lipase, long‐term sustained release	Slow degradation rate, hydrophobic	[79]
	DSPE‐PEG	Bacterial lipase‐triggered degradation for targeted antibiotic release	Limited to infections with lipase‐secreting pathogens	[80]
	Gelatinase‐sensitive peptide G‐1	Sensitive to gelatinase	Potential immunogenicity	[81]

### 3.1 Thermo‐Responsive Materials

Temperature‐responsive materials mainly utilize polymers with a “lower critical solution temperature (LCST)” or an “upper critical solution temperature (UCST).” Among them, polymers with LCST are the most widely used in ophthalmic applications. Below the LCST, these polymers are in a flowable sol state, allowing for administration as eye drops. Upon exposure to the warmer ocular surface (exceeding the LCST), the hydrogen bonds between the polymer and water are disrupted, rapidly transforming the material into a semisolid gel, significantly prolonging drug retention time, and enabling sustained release [82]. Temperature‐sensitive in situ hydrogels are currently one of the most advanced materials in ocular drug delivery.

A range of polymers with LCST have been investigated, including poly (N‐isopropylacrylamide, pNIPAAm), cellulose derivatives, and poloxamer. pNIPAAm is widely studied for drug delivery because it can easily incorporate drugs by dissolving them in the solution at lower temperatures and then forming a gel above its LCST (approximately 32 °C) [83]. Fedorchak et al. developed a gel/microsphere (GMS) eye drop consisting of brimonidine‐loaded microspheres dispersed in a pNIPAAm thermosensitive hydrogel. With a LCST of 33.5 °C, the formulation rapidly formed a gel on the ocular surface after instillation, enabling sustained therapeutic efficacy for up to 28 days following a single administration [84]. To optimize the sol‐to‐gel transition temperature of pNIPAAm gels, Bellotti et al. modified the content and molecular weight of poly (ethylene glycol) (PEG) and achieved a lower LCST (28–29 °C). The system exhibited sustained drug release over 28 days in vitro and a viscosity suitable for eye drop administration while avoiding the risk of reverting to the liquid state under cold conditions [85].

While pNIPAAm is highly effective, its low biodegradability and inadequate mechanical strength require combination with other biomaterials like chitosan [86]. Chitosan (CS), a cationic, biocompatible, and biodegradable polysaccharide formed by deacetylation of chitin, serves as an excellent biomaterial for this purpose. Several studies have prepared ocular DDSs based on CS, such as CS/β‐glycerolphosphate (GP) gels and CS/gelatin/GP [65, 87]. However, the CS/GP hydrogel exhibits opacity and limited solubility at 37 °C. To overcome this, Pakzad et al. developed a thermosensitive hydrogel based on N‐(2‐hydroxy‐3‐trimethylammonium) propyl chitosan chloride (HTCC). Incorporation of sodium hydrogen carbonate into the HTCC/GP system markedly shortened the gelation time from 40 min to 2 min, maintained system transparency, and extended drug release for 1 week [88]. In terms of material modification, Luo et al. investigated the influence of CS degree of deacetylation (DD) on drug release behavior from pNIPAAm‐grafted CS carriers. Their study revealed that the medium‐DD carrier (MC‐PN, 80.3%) provided sustained pilocarpine release over 2 months with optimal biocompatibility [89].

**FIGURE 2:**
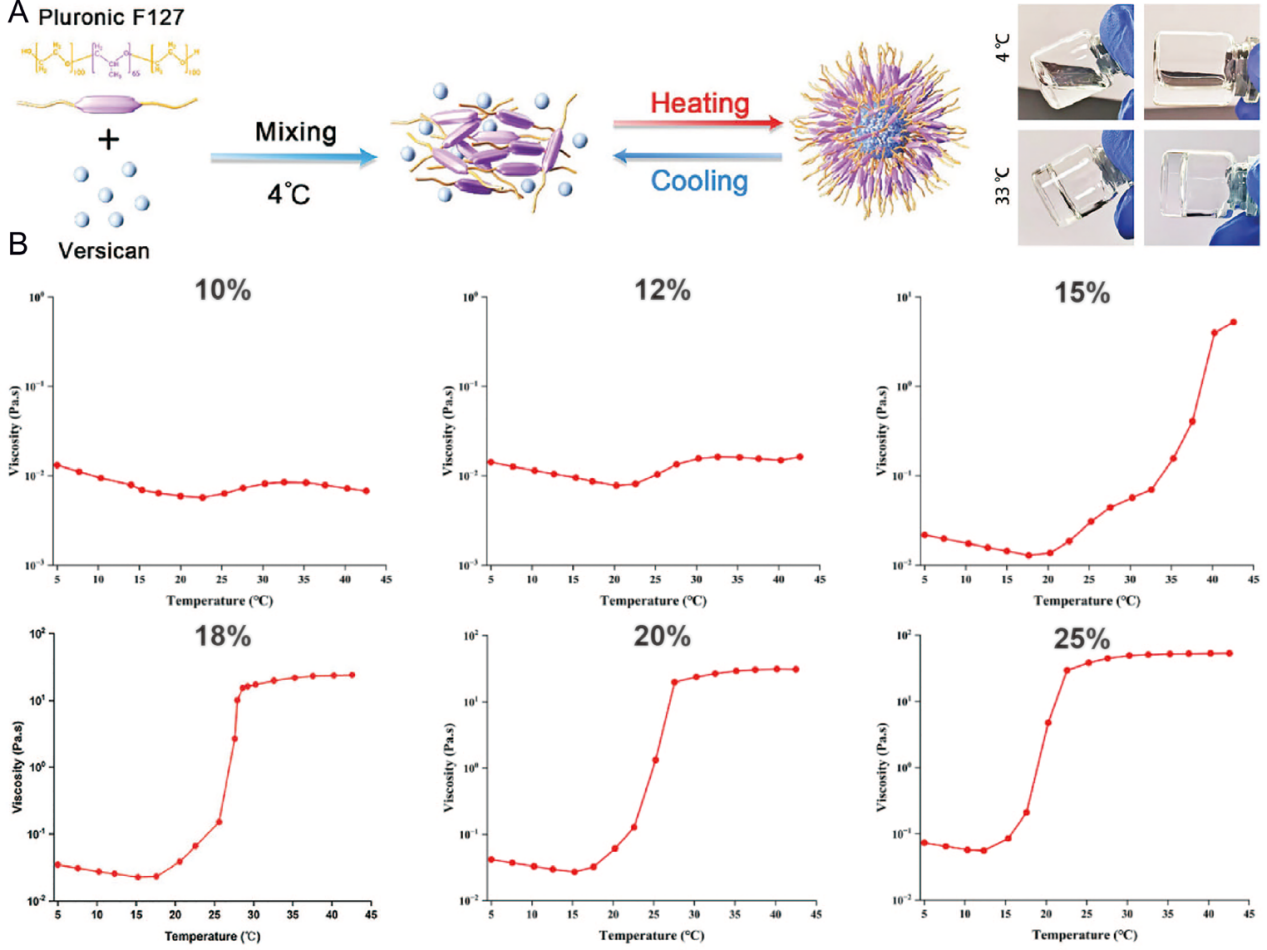
The schematic illustration of thermo‐responsive polymers for drug delivery. (A) The design and fluidity of PF‐127 hydrogel (18% w/w) at 4 and 33 °C. (B) Effect of temperature changes on the viscosity of PF127. Reproduced with permission from ref. [81] Copyright 2024, Wiley.

Poloxamers are amphiphilic polymers with a unique triblock structure, consisting of a central hydrophobic chain of polypropylene oxide (PPO) and two flanking hydrophilic chains of polyethylene oxide (PEO) (PEO‐PPO‐PEO) [90]. Their emulsifying capacity, coupled with thermos‐sensitivity, biocompatibility, and biodegradability, making them ideal for drug delivery. The concentrations of PF‐127 hydrogel were related to their gel time and viscosity. It was found that PF‐127 hydrogel (18% w/w) exhibited a clear liquid at 4 °C, and gel formation occurred at 33 °C (Figure 2A), while 10%, 12%, and 15% w/w could not gel well at 33 °C (Figure 2B). In contrast, the PF‐127 hydrogel (20% and 25% w/w) gelled too quickly and might form uneven gel lumps. Poloxamer 407 (P407, Pluronic F127) and Poloxamer 188 (P188) are commonly used in ocular temperature‐responsive DDSs, where P407 can effectively enhance ocular drug penetration and bioavailability, while P188 is often used to modulate the thermosensitive phase transition temperature [61]. Beyond poloxamers, triblock copolymers with a 3D network constructed from PEG and hydrophobic poly (lactic acid‐co‐glycolic acid) (PLGA) have been extensively studied. PLGA‐PEG‐PLGA (PPP) has excellent encapsulation capability, can improve the solubility and stability of lipophilic drugs, protect the encapsulated drugs from rapid degradation, and enhance drug bioavailability [62].

### 3.2 pH‐Responsive Materials

pH‐responsive materials are commonly constructed with ionizable functional groups, such as carboxylic acids (−COOH) or amines (−NH_2_), which undergo protonation or deprotonation in response to changes of pH. This change alters the electrostatic interactions and hydrophilicity‐hydrophobicity balance within the polymer; induces structural transitions, such as swelling, contraction, gelation, or degradation; and ultimately triggers the release of encapsulated drugs. Commonly used pH‐responsive polymers include poly (acrylic acid) (PAA), chitosan, Eudragit S‐100, polyethylenimine (PEI), and Schiff base linkages [91, 92].

Among these, carbopol, a cross‐linked PAA polymer, is one of the most extensively studied polymers for ophthalmic DDSs, and it undergoes a sol–gel transition as the pH rises more than 5.5 pKa value. In acidic environments, the carboxylic groups of carbopol remain dissociated, leading to a flexible coiled polymer structure. Upon exposure to the slightly alkaline physiological environment, the carboxylic groups ionize, imparting a negative charge along the polymer backbone. Subsequent electrostatic repulsion between the anionic polymer and the negatively charged tear fluid induces polymer uncoiling, swelling, and ultimately, gel formation [93]. However, due to its acidic nature, carbopol can cause ocular irritation, which often necessitates its combination with other polymers like hydroxypropyl methylcellulose (HPMC) to reduce its concentration and mitigate adverse effects [94, 95].

Similarly, chitosan (CS) is a cationic pH‐responsive polymer, which also undergoes a sol–gel transition around pH 6.5 as the environment shifts from slightly acidic to neutral. Its pH‐responsive behavior originates from the protonation and deprotonation of the primary amino groups (−NH_2_). When the pH value is below its pKa of approximately 6.5, these amino groups protonated to −NH_3_^+^, generating enhanced electrostatic repulsion and hydrophilicity, ultimately leading to dissolution into a transparent sol. Conversely, at physiological pH (e.g., ~7.4 on the ocular surface), the amino groups deprotonate, resulting in reduction in electrostatic repulsion and formation of gel [96]. For instance, when a CS solution dissolved in a mild acid is administered as eye drops or an injection, it spontaneously forms a gel upon contact with the neutral pH of the ocular surface, thereby significantly prolonging drug residence time at the target site [97].

Eudragit S‐100 represents a distinct class of an anionic copolymer synthesized from methacrylic acid and methyl methacrylate in an approximate molar ratio of 1:2 [64]. Its pH‐responsive behavior stems from the ionization state of the carboxyl groups on the polymer side chains. In acidic or neutral environments (below pH 7.0), the carboxyl groups remain protonated and uncharged, keeping the polymer chains in a collapsed and insoluble state. When the pH rises to 7.0 or above, the carboxyl groups undergo deprotonation, forming negatively charged carboxylate anions [69]. This ionization introduces strong electrostatic repulsion between polymer chains, promotes hydration, and leads to dissolution. For instance, Maulvi et al. engineered a pH‐triggered drug release system by incorporating Eudragit S‐100 NPs into cyclosporine‐loaded CLs. The NPs remained intact and retained the drug at neutral or acidic pH (below 7.0). However, upon exposure to the elevated pH characteristic of the DED (pH > 7.8), the Eudragit matrix dissolved, thereby releasing the therapeutic payload [98].

Another strategy involves pH‐sensitive linkages such as the Schiff base, which remains stable under neutral conditions but quickly hydrolyzes under weakly acidic conditions. Li et al. designed pH‐responsive NPs using an amphiphilic polymer‐drug conjugate, PEG‐Schiff‐vancomycin (PEG‐Schiff‐Van). These NPs exhibited programmed drug release, enabling an initial burst to rapidly eliminate bacteria, followed by sustained pH‐responsive release to maintain long‐term antibacterial efficacy [99].

### 3.3 Ion‐Responsive Materials

Ion‐responsive materials refer to a class of functional polymers capable of interacting with cations such as K^+^, Na^+^, or Ca^2+^, etc., leading to sol–gel transitions through ion‐induced cross‐linking, which alters their solubility or chain conformation. Commonly used ion‐responsive polymers in ocular DDSs include gellan gum, κ‐carrageenan, and alginate.

Gellan gum is an anionic linear polysaccharide composed of glucose, glucuronic acid, and rhamnose [100]. When triggered by cations in the tear fluid (e.g., Na^+^, Mg^2+^), these ions electrostatically screen the negative charges between polymer chains and form ionic bridges with the carboxyl groups of glucuronic acid residues. This induces a conformational rearrangement of the polymer chains from random coils into ordered double helices, which further aggregate via noncovalent interactions, forming a 3D network that transforms the solution into a gel. The ion‐triggered gelation is highly sensitive and fragile, with a decrease in ionic concentration leading to gel disintegration [67]. Based on this mechanism, gellan gum can undergo in situ gelation upon instillation into the eye via electrolytes in the tear film, thereby prolonging ocular residence time and controlling drug release [64, 97].

κ‐Carrageenan is a linear polysaccharide with anionic sulfate esters [101]. Its gelation is highly dependent on the presence of potassium ions (K^+^). The K^+^ ions not only shield interchain electrostatic repulsion but also specifically bridge sulfate groups between adjacent helices via stable “potassium bridges,” promoting further aggregation of the double helices into a brittle 3D gel network [102].

**FIGURE 3:**
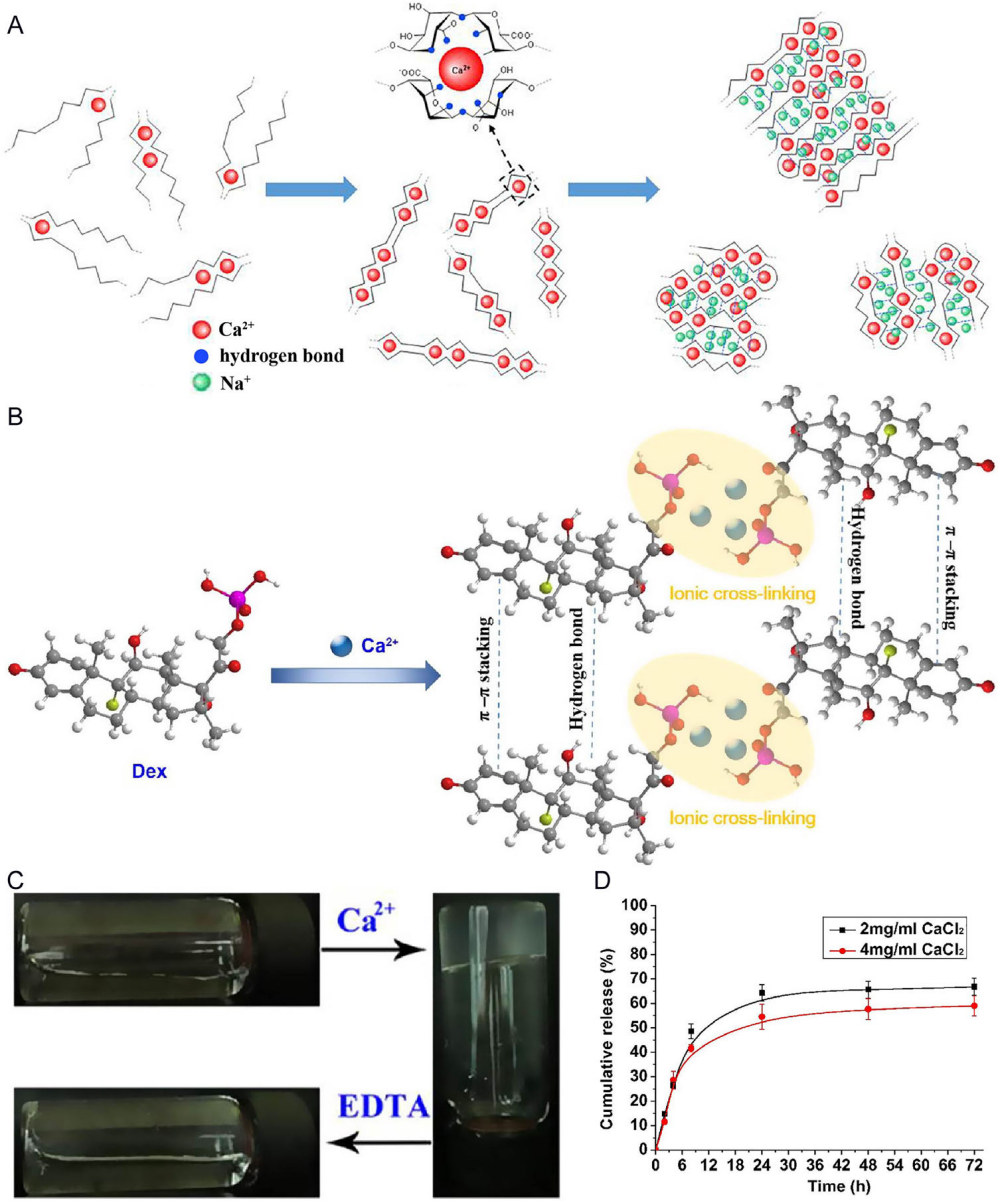
Mechanisms of ion‐sensitive polymers for drug delivery. (A) Schematic of the mechanism in gelation of alginate. Reproduced with permission from ref. [105] Copyright 2021, Elsevier. (B) Formation of Dex supramolecular hydrogel. (C) Images of the Ca^2+^ and EDTA‐induced sol–gel‐sol transition. (D) The cumulative drug release from Dex hydrogel formed by 2 mg/ml and 4 mg/ml CaCl_2_. Reproduced with permission from ref. [70] Copyright 2017, Elsevier.

Alginate exhibits a notably different ion‐responsive mechanism. As an unbranched anionic polysaccharide derived from brown algae, it is composed of alternating guluronic acid (G) and mannuronic acid (M) residues. Although alginate can form gels through cross‐linking with various cations such as H^+^, Ca^2+^, Zn^2+^, and Fe^2+^, Ca^2+^‐induced gelation represents its most functionally significant characteristic [103, 104]. Ca^2+^ ions fit into the stereochemical cavities formed by adjacent G blocks in an “egg‐box” manner, coordinating precisely with the carboxylate groups, forming strong ionic bridges between polymer chains and then directly constructing a 3D network (Figure 3A). This process occurs predominantly between G blocks, yielding gels with high mechanical strength and often irreversible character [105].

It is noteworthy that Wu et al. innovatively employed an ionic cross‐linking strategy to construct a supramolecular hydrogel composed of dexamethasone sodium phosphate (Dex) and calcium ions, thereby avoiding complex synthesis and the need for exogenous carriers (Figure 3B) [70]. Gelation occurred rapidly with increasing concentrations of CaCl_2_ and Dex, while incubation of the hydrogel with an aqueous EDTA solution, an effective Ca^2+^ chelator, led to its dissolution into a transparent solution (Figure 3C). And the drug release rate of the hydrogel could be modulated by varying the calcium ion concentration (Figure 3D).

### 3.4 ROS‐Responsive Materials

ROS‐responsive materials represent a class of intelligent delivery systems designed to specifically recognize pathologically elevated levels of ROS, such as H_2_O_2_ and ·OH, and undergo controlled structural changes to enable targeted drug release. Under oxidative stress conditions such as inflammation or cancer, the elevated ROS triggers cleavage or transformation of ROS‐sensitive chemical motifs, leading to carrier degradation or property alteration, thereby facilitating site‐specific drug release [106]. The commonly used ROS‐sensitive chemical motifs including thioether, selenide, telluride, and arylboronic ester groups.

#### 3.4.1 Sulfur‐Containing ROS‐Responsive Materials

**FIGURE 4:**
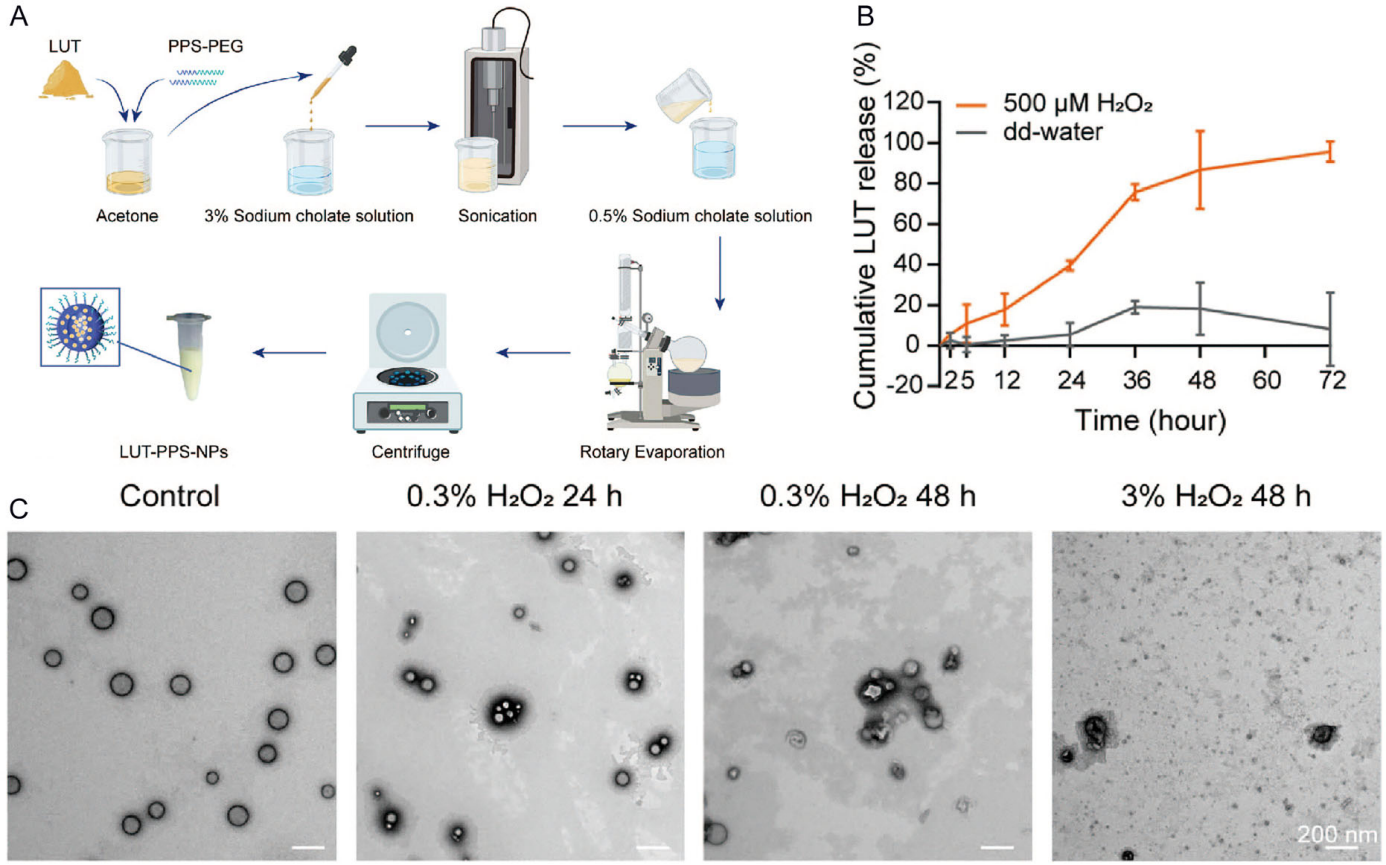
The schematic illustration of ROS‐responsive DDSs. (A) Schematic Illustration of the preparation of ROS‐responsive NPs using polymer PEG–PPS. (B) The cumulative release behavior in dd‐water or 500 μM H_2_O_2_. (C) Electron micrographs of PPS‐NPs degradation after H_2_O_2_ treatment. Reproduced under the terms of the CC‐BY‐NC license, ref. [112] Copyright 2025, Taylor & Francis.

Sulfur‐based polymers including thioketal, poly (propylene sulfide) (PPS) and 2‐(methylthio)ethyl methacrylate (MEMA) were widely studied in DDS [71, 107–110]. Thioketal (TK), containing a —S—C—S— bond, can cleave under elevated ROS in CNV lesions, yielding ketones and thiols without generating acidic byproducts, thereby ensuring good biocompatibility [111]. PPS, a hydrophobic polymer with thioether groups, can be oxidized rapidly and disintegrated into hydrophilic sulfoxides and sulfones in the presence of high H_2_O_2_ concentrations, resulting in carrier disintegration and drug release. NPs or micelles prepared from the amphiphilic block copolymer PEG‐PPS are particularly valued for their water solubility and biodegradability and have been extensively applied in anticancer immunotherapy and anti‐inflammatory therapies (Figure 4) [112].

#### 3.4.2 Selenium‐Containing ROS‐Responsive Materials

Selenium‐containing polymers represent another important category of ROS‐responsive materials [113, 115]. The selenium (Se) atom exhibits higher oxidative sensitivity and a lower redox potential compared to its sulfur analogs, allowing faster drug release even at relatively low ROS levels. These materials also display good biocompatibility and degradability, showing considerable potential in anti‐inflammatory and antioxidant therapies [74].

#### 3.4.3 Arylboronic Ester‐Based ROS‐Responsive Materials

Arylboronic ester‐based polymers constitute a third major type of ROS‐responsive carrier. The boronic ester bond is susceptible to oxidative hydrolysis in the presence of ROS, yielding hydrophilic boronic acid and phenol derivatives, which induces carrier disassembly and targeted drug release [75, 116]. Niu et al. conjugated 4‐carboxyphenylboronic acid pinacol ester (EB) with glycol chitosan (GC) and the antifungal drug voriconazole (VOR) to construct a nanocarrier system (GC‐EB‐VOR) [117]. This system exhibited dose‐dependent ROS‐responsive behavior and efficient VOR release upon H_2_O_2_ treatment. By responding to ROS at the infection site and thereby enabling precise releasing of VOR, GC‐EB‐VOR effectively reduced inflammation and scavenged ROS, demonstrating promising antifungal efficacy.

### 3.5 Enzyme‐Responsive Polymers

**FIGURE 5:**
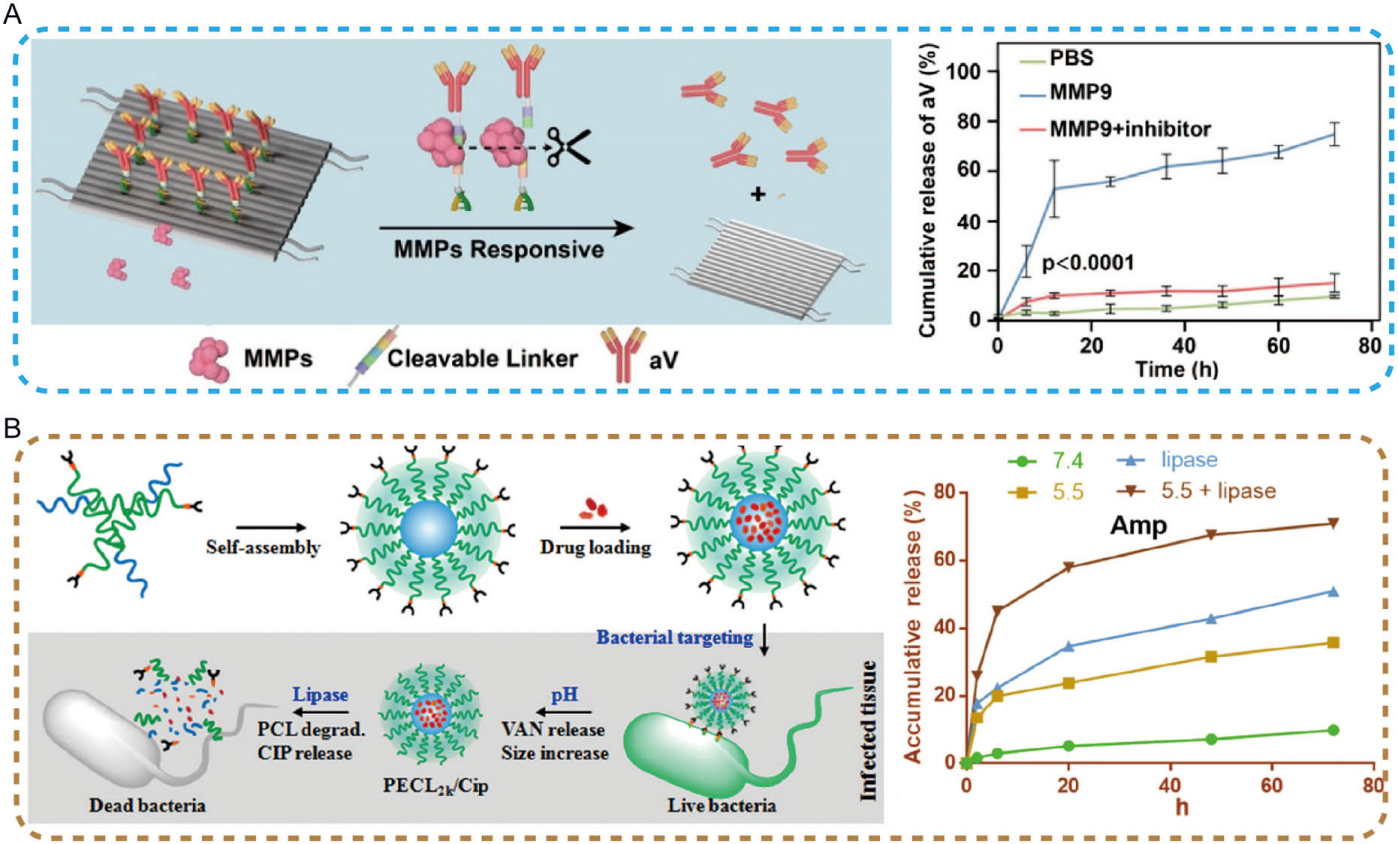
The schematic illustration of enzyme‐responsive DDSs. (A) Schematic illustration of MMP9 responsive carrier and cumulative release behavior treated with MMP9. Reproduced with permission from ref. [119] Copyright 2023, American Chemical Society. (B) Schematic illustration of lipase responsive carrier and cumulative release behavior under different conditions. Reproduced with permission from ref. [42] Copyright 2018, American Chemical Society and ref. [120] Copyright 2023, Elsevier.

Enzyme‐responsive polymers represent a class of intelligent material that achieve controlled drug release through enzyme‐mediated reactions. The core design strategy involves incorporating substrate sequences of specific enzymes, such as MMPs into the material backbone as cleavable cross‐linkers or degradable segments. Under physiological conditions, the material structure remains intact due to the low expression of the target enzymes. However, when the carrier reaches pathological sites with overexpressed enzymes (e.g., MMPs, lipase, gelatinase), the substrate sequences are specifically recognized and cleaved, leading to the disintegration of the material network and subsequent targeted drug release [118]. For example, Wu et al. designed an MMP‐responsive nanocarrier for anti‐VEGF antibody delivery (aV). The system efficiently released aV when treated with MMP‐9, whereas the MMP‐9 inhibitor GM6001 strongly suppressed this proteolytic process (Figure 5A) [119].

Several DDSs have been engineered to achieve on‐demand antimicrobial release by responding to bacterial‐secreted enzymes such as hyaluronidase, lipase, and gelatinase at the infection site [121]. For instance, using the presence of lipase in infected microenvironments, lipase‐sensitive polymers like PCL and 1,2‐distearoyl‐sn‐glycero‐3‐phosphoethanolamine‐PEG (DSPE‐PEG) have been utilized to selectively targeted bacterial infection sites [42, 77, 120]. Chun et al. developed an infection‐targeting nanomicelles (NMs) system with lipase‐responsive drug release capabilities [120]. As shown in Figure 5B, the accumulative release of ampicillin (Amp) under pH 5.5 was nearly 40% over 72 h, compared with less than 10% under physiological conditions (pH 7.4), indicating an acceleration of Amp release at acidic environment. In the presence of both acidic pH and lipase, the cumulative release increased dramatically to approximately 70% at 72 h.

Leveraging the overexpression of ALP in autoimmune uveitis, Li et al. developed an ALP‐responsive supramolecular hydrogel for the co‐delivery of dexamethasone sodium phosphate (Dex) and adalimumab (ADA). The system provides enzyme‐triggered release by leveraging ALP‐mediated dephosphorylation of Dex to liberate the active Dex. It was demonstrated that a single intravitreal injection of the hydrogel markedly alleviated intraocular inflammation, outperforming the efficacy of combined ADA and Dex solution [78].

## 4 Preparation Methods and Characterization Techniques of Stimulus‐Responsive DDSs

The preparation methods and characterization techniques of stimulus‐responsive systems depend on both the responsive polymers and the delivery forms. With advances in materials science, a single responsive polymer can be processed into multiple delivery forms to meet different clinical requirements. In this section, we focused on three representative delivery systems: hydrogels, microneedles (MNs), and nanocarriers. For each delivery form, representative preparation methods and characterization technique were summarized.

### 4.1 Preparation Method

#### 4.1.1 Hydrogels

Hydrogels are 3D hydrophilic polymer networks that can be prepared via either physical crosslinking or chemical crosslinking. Physical crosslinking relies on physical forces such as hydrogen bonding, hydrophobic interactions, and chain entanglements. The process is typically reversible and offers better biocompatibility. While chemical crosslinking forms permanent networks through covalent bonds, as seen in the preparation of poly(vinyl alcohol) hydrogels using glutaraldehyde [121]. It generally provides higher mechanical strength but may involve toxic crosslinking agents or initiators. In this study, the thermosensitive, pH‐sensitive, and ion‐sensitive hydrogels are all formed by physical crosslinking. For example, Li et al. developed a thermosensitive DDS named SEED, which was prepared by physically mixing versican with PF‐127 at 4 °C. Briefly, PF‐127 powder was dissolved in precooled sterile deionized water, followed by the addition of versican solution [81].

#### 4.1.2 MNs

MNs offer a minimally invasive approach to bypass ocular surface barriers and deliver therapeutics directly into corneal tissue. Based on their preparation methods and characteristics, MNs can be categorized into several types, including solid MNs, hollow MNs, coated MNs, dissolving MNs, and hydrogel MNs [80]. Solid and hollow MNs are typically fabricated from metals or silicon using photolithography and electrochemical etching techniques. Their application involves a two‐step process: First, the MNs create microchannels in the cornea, followed by drug application to the treated area [79]. Coated MNs, on the other hand, employ dip‐coating or spray‐coating techniques to apply formulations containing proteins. Dissolving and hydrogel MNs are generally prepared by casting polymer blends into molds under centrifugation or vacuum, followed by solidification through drying or UV curing [122]. For instance, Chen et al. developed a bilayer hydrogel MN patch for the treatment of corneal injury‐associated bacterial keratitis, combining pH‐responsive MN tips with UV‐triggered backing layer. The MN tips were fabricated by casting a solution containing 12% methacrylated hyaluronic acid (HAMA), 1% type I collagen (COL I), 0.05% decellularized corneal extracellular matrix (ECM), and PPAQ liposomes (pH‐responsive, azithromycin and quercetin co‐loaded liposomes) into a polydimethylsiloxane (PDMS) mold, followed by drying in a desiccator for 2 days. The backing layer was formed by casting a second solution comprising 12% HAMA, 1% COL I, 0.05% ECM, and 0.1% lithium phenyl‐2,4,6‐trimethylbenzoylphosphinate as a photoinitiator [92].

#### 4.1.3 Nanocarriers

Nanocarriers offer a versatile platform to encapsulate therapeutic agents, enhance drug solubility, and overcome ocular absorption barriers. Common nanocarrier used in ocular drug delivery includes polymeric NPs, liposomes, nanogels and NMs. Different preparation methods are employed to fabricate these nanocarriers, each influencing particle size, drug loading efficiency, and release kinetics. Polymeric NPs are typically synthesized via ionic gelation, solvent diffusion, or nanoprecipitation techniques [123]. For instance, miltefosine‐loaded chitosan NPs were fabricated by ionic gelation. Specifically, chitosan was dissolved in acetic acid solution and then crosslinked by addition of tripolyphosphate under stirring, followed by freeze‐dried to form stable NPs [59]. Similarly, cyclosporine‐loaded Eudragit S100 NPs were prepared by a quasi‐emulsion solvent diffusion method, where the drug and polymer are dissolved in acetone and injected into an aqueous PF127 solution under high‐speed homogenization, followed by solvent evaporation and freeze‐drying [98].

Liposomes are commonly prepared using thin‐film hydration followed by sonication or extrusion to control vesicle size and lamellarity, with smaller unilamellar vesicles showing improved ocular penetration [124]. For example, azithromycin and quercetin co‐loaded liposomes (PPAQ Lip) were prepared by first synthesizing azithromycin‐loaded liposomes via thin‐film hydration using soy phospholipid and cholesterol, followed by electrostatic adsorption of PEI‐PBA onto the liposome surface, and finally conjugation of quercetin through dynamic boronic ester bonds to obtain pH‐responsive liposomes [92].

NMs are generally formed through nanoprecipitation or dialysis, where amphiphilic polymers self‐assemble into micellar structures with hydrophobic cores for drug encapsulation. A representative example is the synthesis of PEG‐b‐PPS block copolymer, where PEG thioacetate is first synthesized and then used as a macroinitiator for polymerization of propylene sulfide. The copolymer self‐assembles into micelles in aqueous solution, encapsulating hydrophobic drugs such as celastrol within the hydrophobic PPS core. The drug loading is achieved by dissolving the polymer and drug in tetrahydrofuran, followed by dropwise addition into water under ultrasonication and removal of organic solvent by rotary evaporation [125].

Nanogel preparation involves either chemical crosslinking (using toxic crosslinkers such as glutaraldehyde and phthalaldehyde) or physical crosslinking (via electrostatic interactions, and hydrogen bonding), which determines the nanogel's stability and responsiveness to stimuli. Alternatively, supramolecular host–guest interactions have emerged as a viable approach for nanogel fabrication, offering strong binding affinity and avoiding toxic crosslinkers [126]. For instance, Xiang et al. developed a ROS‐responsive nanogel (DEX@INHANGs) for CNV therapy. The nanogel was constructed via host‐guest complexation between cyclodextrin conjugated on hyaluronic acid and adamantane (ADA) modified on an 8‐arm PEG‐thioketal polymer. The thioketal (TK) linkage served as a ROS‐responsive moiety, ensuring controlled dexamethasone release under oxidative stress conditions. Additionally, integrin β1 fusion protein was covalently conjugated to the nanogel surface to prolong corneal retention for over 8 h. The resulting nanogels exhibited spherical morphology, negative surface charge, and stability for at least 7 days, demonstrating their potential as an effective ocular drug delivery platform [127].

### 4.2 Characterization Techniques

The physicochemical properties of MR‐DDSs including chemical bonds, morphology, surface charge, sol–gel transition conditions, stimulus‐responsive drug release performance, and biocompatibility are closely related to their application and therapeutic efficacy. In this section, we summarized common characterization techniques based on their purpose: chemical structure confirmation, physicochemical property evaluation, stimulus‐responsive performance, and biocompatibility assessment.

#### 4.2.1 Chemical Structure Characterization

Chemical structure confirmation typically employs proton nuclear magnetic resonance spectroscopy (^1^H NMR) to verify polymer conjugation and confirm successful introduction of functional groups, such as Schiff base formation (detected by characteristic imine protons around δ 8.4 ppm) or aldehyde group modification (e.g., *δ* 9.59 ppm for –CHO) [128, 129]. Fourier transform infrared (FTIR) Spectroscopy is used to identify characteristic bonds, such as amide bond formation (IR bands located at around 1662 cm^−1^) and C=O (IR bands located at 1725 cm^−1^) [117, 128]. X‐ray photoelectron spectroscopy (XPS) provides complementary information on elemental composition such as boron (from boronic esters) or selenium (from selenides in ROS‐responsive polymers) [130]. These techniques collectively ensure successful material synthesis prior to functional testing.

#### 4.2.2 Physicochemical Characterization

Particle size (PS) and polydispersity index (PDI), essential parameters affecting nanocarrier stability and cellular uptake, are mainly evaluated by dynamic light scattering (DLS) [131]. Electron microscopy approaches, including transmission electron microscopy (TEM) for internal morphology and scanning electron microscopy (SEM) for surface topography, are used to visualize NPs shape and hydrogel microstructure [132]. Zeta potential measurements assess surface charge, which influences colloidal stability and mucoadhesive interactions with negatively charged ocular surface [98]. Rheometer analysis determines sol–gel transition temperatures, storage/loss moduli (G′ and G″), and shear‐thinning behavior, which are critical for predicting in situ gelation, mechanical integrity, and ease of administration [133]. UV–vis spectrophotometry is generally used to evaluate optical transparency, quantify drug loading, and encapsulation efficiency [134].

#### 4.2.3 Stimulus‐Responsive Performance Characterization

Stimulus‐responsive behavior is typically evaluated using in vitro release studies under simulated physiological and pathological conditions. The dialysis bag method is commonly employed: The formulation is placed in a dialysis bag and immersed in PBS containing or lacking a triggering factor, such as H_2_O_2_ for ROS‐responsive systems), or MMP‐9 for enzyme‐responsive systems [133, 119]. Complementary techniques include DLS to monitor stimulus‐induced particle size changes indicating carrier degradation or swelling [132].

#### 4.2.4 Biocompatibility and Safety

Biocompatibility assessment is essential for evaluating clinical translational potential. In vitro cytotoxicity is determined using CCK‐8 or MTT assays on corneal epithelial cells, and often supplemented by live/dead staining for visual confirmation of cell viability [135]. In vivo ocular safety is assessed via the Draize test (scoring corneal opacity, conjunctival redness and chemosis) and fluorescein sodium staining to detect corneal epithelial defects [136]. Histological examination (H&E staining) of cornea, conjunctiva, and major organs (heart, liver, spleen, lung, kidney) evaluates tissue damage, inflammation, or foreign body reactions [125].

## 5 Applications of Stimulus‐Responsive DDSs in Major Ocular Surface Diseases

In recent years, stimulus‐responsive materials have shown remarkable progress in the treatment of various ocular diseases. This section summarizes the pathological microenvironmental changes and applications of such materials in several common ocular surface disorders, including DED, IK, and CNV (Table 3).

**TABLE 3:** Application of microenvironment‐responsive DDSs in major ocular surface disease.

Disease	Stimulus	Drug carrier	Polymer	Cargos	**Drug loading efficiency**	Release duration	Ocular residence time	Penetr‐ation depth	Therapeutic efficacy		Ref.
DED	Temperature	Hydrogel, NPs	P407+acetylated PEI‐modified PLGA for NPs	RSV	78.3 ± 4.9%	72 h	—	—	Reduced ROS and MDA; restored SOD and CAT activities; improved mitochondrial respiration; upregulated SIRT1; suppressed inflammation		Luca et al. [132]
	Temperature	Hydrogel	P407	CDs	—	4 h	≥90 min	—	Eliminated ROS and DHE signals; reduced TUNEL‐positive cells; increased goblet cells; promoted tear secretion		Yang et al. [137]
	Temperature	Hydrogel, NPs	Aldehyde‐functionalized P407	Cu2‐x Se	—	72 h	≥60 min	—	Restored corneal epithelium thickness; increased goblet cells; promoted tear secretion	Ou et al. [129]	
	Temperature	Hydrogel	P407/P188/HPMC	C‐dots	—	—	120 min	—	Restored corneal epithelium thickness and goblet cells	Wei et al. [138]	
	Temperature	Hydrogel	pNIPAAm/gelatin	EGCG	Nearly 100%	72 h	—	—	Reduced DPPH and ROS; restored goblet cells and MUC5AC expression	Luo et al. [139]	
	Temperature	Hydrogel, micelles	MPOSS/PEG/PPG	FK506	1MPEP, 93.4% 2MPEP, 92.8%	14 days	—	—	Restored corneal epithelium and goblet cells; reduced MMP‐3/9 expression and CD4+ T cell infiltration		Han et al. [133]
	pH	CLs	CAP	CsA	—	24 h	24 h	—	Increased tear volume, TBUT and goblet cells; decreased apoptotic cells		Kim et al. [136]
	pH	CLs, NPs	Eudragit S100	CsA	ENP 1:1: 78.6%; ENP 1:2: 90.5%; ENP 1:3: 93.1%	ENP 1:1: 156 h; ENP 1:2: 120 h; ENP 1:3: 72 h	14 days	—	—		Maulvi et et al. [98]
	Ion	Hydrogel	Gellan gum	TFC	—		≥60 min	—	Restored goblet cells and tear volume; inhibited inflammation		Chen et al. [128]
	Ion	Hydrogel	KFQ12	V1‐Cal peptide	—	over 7 days	>40 min	—	Promoted corneal defect recovery, TBUT (1.3→5.5 s), tear volume (1.4→4.6 mm); reduced TUNEL		Ding et al. [140]
	ROS	MN patch	EGCG	CsA	—	>48 h	48 h	600 µm	Elevated tear secretion (2.6→4.3 mm), goblet cells (8.3→18.5); restored lacrimal gland, downregulated Th1/Th17, TNF‐α, IL‐6, NO, iNOS, COX‐2		Mu et al. [141]
	ROS	NMs	PEG‐b‐PPS	Cel	56.24%	—	—	—	Promoted tear volume and goblet cells; downregulated TLR4/MyD88/NF‐κB;		Cui et al. [125]
IK	Temperature	Hydrogel	Chitosan/β‐glycerophosphate	Moxifloxacin, gentamicin	—	Over 24 h	—	—	completely inhibited S. aureus growth		Mohammed et al. [142]
	Temperature	Hydrogel, NLC	P407/HPMC	Sertaconazole	89.97%	Over 24 h	—	—	Higher antifungal activity against Candida albicans than free drug		Tavakoli et al. [131]
	Temperature	Hydrogel	PLGA‐PEG‐PLGA	Voriconazole	—	7 days	7 days	—	–		Pereira et al. [62]
	Temperature	Hydrogel, NPs	P407+gelatin‐PAA NPs	Oxytetracycline	—	over 24 h	—	—	Comparable to commercial ointment (Terramycin) in Pseudomonas keratitis model; complete cure at 7 days		Abbas et al. [135]
	pH	CLs	ACPA	Van	—	8 h	—	—	Excellent bactericidal against S. aureus and S. epidermidis; reduced corneal edema and inflammation		Guo et al. [130]
	pH	CLs	CNF	LFX	—	24 h	–—	—	–		Siripongpreda et al. [72]
	pH	MNs	PEI	Quercetin, azithromycin	—	72 h	11 days	210 μm	Superior to daily eye drops (CIP, AZI); single application achieved effective bacterial eradication and corneal regeneration		Chen et al. [92]
	Ion	Hydrogel	Deacylated gellan gum/κ‐carrageenan	Econazole	—	24 h	72 min	—	Effective against C. albicans, A. fumigatus, Paecilomyces		Victoria et al. [143]
	Ion	Hydrogel	Gellan gum	Gatifloxacin	—	5 days	—	—	Better improvement in S. aureus keratitis than marketed drops (Gatiquin); less frequent dosing (twice vs. four times daily)		Kesavan et al. [144]
	ROS	Nano‐carrier	EB	VOR	96%	>50 h	—	—	Reduced ROS and inflammation in Fusarium ketatitis		Niu et al. [117]
	Temperature/pH	Hydrogel, NPs/liposomes/NLC	P127/chitosan	PI	PI‐CS NPs: 77.25%; PI‐LPs: 94.36%; PI‐NLC: 49.82%	Over 24 h	—	—	PI‐CS NPs gel: 92% ameba inhibition (vs. 50% for commercial drops); best among formulations		Basant et al. [63]
CNV	Temperature	Hydrogel	PECG	Ava	—	28 days	21 days	—	Superior anti‐angiogenic efficacy vs. Ava solution in rabbit CNV model		Xu et al. [145]
	Temperature	Hydrogel	P407, EPL	BMP4	—	>10 h	—	—	More effective than BMP4 solution in inhibiting CNV; downregulated VEGF and MMP9 expression		Xu et al. [146]
	Temperature	Hydrogel	P407, OSA	BMP4	—	>72 h	—	—	Significantly reduced CNV length and area; superior to BMP4 solution; repaired corneal epithelial apical junctional complexes		Nan et al. [147]
	Temperature	Hydrogel	OCMC, P407‐NH_2_	BMP4	—	>48 h	—	—	Significantly inhibited CNV, reduced inflammatory cell infiltration and promoted cell proliferation		Yang et al. [148]
	ROS	NPs	ROS‐TK‐5+DSPE‐PEG	Anti‐VEGF siRNA	>90%	>10 h	—	—	Significantly reduced CNV area and vessel length in alkali burn model; downregulated VEGF, VEGFR2, and pro‐angiogenic cytokine		Liu et al. [149]
	ROS	CLs	MEMA	Dex	—	7 days	45.83 h	—	CNV inhibition rate 76.53% on day 14; downregulated VEGF, MMP‐2, MMP‐9; reduced ROS		Sun et al. [134]
	ROS	Nanogel	TK	Dex	55.1%	70 h	Over 8 h	—	Once‐daily topical application effectively suppressed CNV		Ding et al. [126]
	ROS/temperature	hydrogel	Se‐PEG‐PPG	Feno	—	—	—	—	Significantly inhibited CNV; scavenged ROS; downregulated VEGF, MMP‐9, IL‐1β, IL‐6, TNF‐α; activated NRF2/HO‐1 pathway;		Fan et al. [74]

### 5.1 DED

DED is a prevalent global ocular disorder primarily caused by reduced tear secretion, tear film instability, and increased tear evaporation, leading to symptoms such as dryness and irritation [150]. It is estimated that the global prevalence of DED has reached 34.6% [151]. Research indicates that the pathogenesis of DED is closely associated with multiple pathophysiological unbalances in the ocular surface microenvironment. For instance, tear film instability accelerates local evaporation, causing the ocular surface temperature in DED patients to drop by approximately 0.1–0.2 °C within seconds [152]. Furthermore, excessive tear evaporation elevates tear osmolarity, which can induce overproduction of ROS [153, 154]. ROS activates the TRPM2 channel, subsequently triggering the NLRP3 inflammasome and converting inactive IL‐1β into its active form, ultimately leading to ocular surface inflammation [155]. Studies have also shown that hyperosmolarity upregulates TNF‐α expression in corneal epithelial cells and stimulates the synthesis and release of multiple MMPs, including MMP‐1, MMP‐3, MMP‐9, and MMP‐13 [156, 158].

Current clinical management of DED remains predominantly pharmacological, relying on agents such as artificial tears, cyclosporine ointment, and corticosteroids. These treatments typically require frequent daily administration or long‐term use, which not only imposes a burden on patients’ daily lives but may also lead to issues such as local irritation and reduced tolerance. Therefore, the development of novel MR‐DDSs that can reduce dosing frequency and enhance therapeutic efficacy is of significant importance.

#### 5.1.1 Sustained Drug Release via Thermo‐/pH‐/Ion‐Sensitive Systems for DED

**FIGURE 6:**
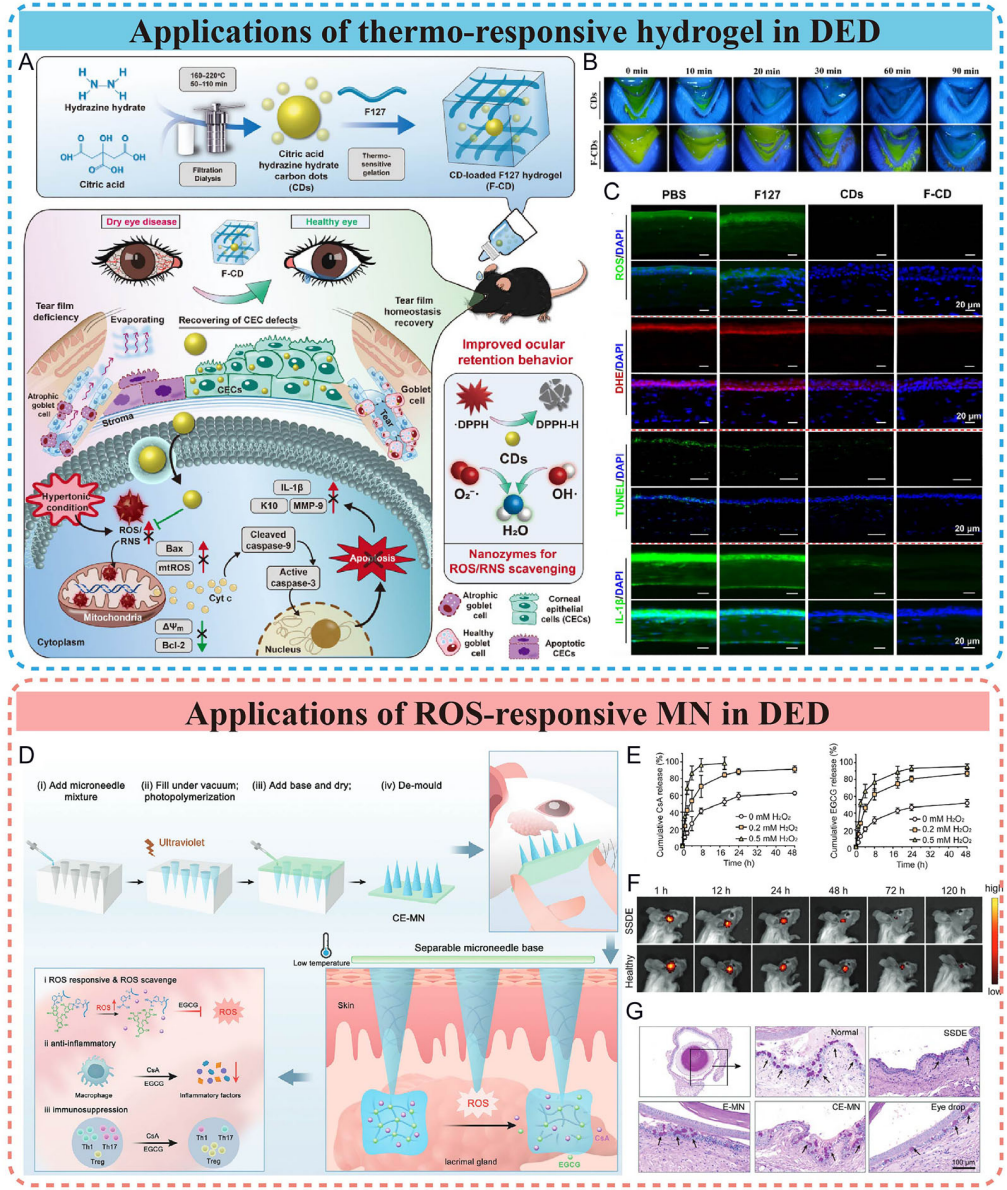
Applications of MR‐DDSs in DED. (A) Schematic illustration of the synthesis and therapeutic mechanism of F‐CDs in DED. (B) Comparison of ocular surface retention time. (C) Evaluations of the antioxidative effects. Reproduced under the terms of the CC BY‐NC License 4.0, ref. [137] Copyright 2025, American Association for the Advancement of Science. (D) Fabrication process and ROS‐responsive mechanisms of CE‐MN patches. (E) Concentration‐dependent release of CsA and EGCG from CE‐MN system in response to H_2_O_2_. (F) Time‐course in vivo fluorescence images of CE‐MN. (G) Representative PAS stained conjunctival sections. Reproduced under the terms of the CC BY License 4.0, ref. [141], Copyright 2024, Wiley‐VCH GmbH.

Thermo‐/pH‐/ion‐sensitive hydrogels are widely used in DED treatment due to their ability to undergo a sol–gel transition at ocular surface, significantly prolonging drug residence time on cornea. Among these, P407, a member of the Pluronics family of triblock copolymers, is widely employed as a thermoresponsive, water‐soluble pharmaceutical excipient [159]. Yang et al. developed an innovative thermos‐sensitive, metal‐free carbon nanodot (CD) hydrogel, termed F‐CD hydrogel (Figure 6A). As shown in Figure 6B, the majority of the CD solution was eliminated within 10 min after administration, whereas the F‐CDs hydrogel remained on the ocular surface for up to 90 min. This hydrogel alleviated DED symptoms through ROS scavenging, anti‐inflammatory activity, and antiapoptotic effects (Figure 6C) [137]. However, high concentrations of P407 are required for adequate gelation, raising concerns about ocular irritation. To address this, Wei et al. incorporated P188 and HPMC E50 into a P407‐based formulation (21% P407, 1.0% P188, 1.0% HPMC E50), achieving rapid gelation (3–4 s) while mitigating irritation. Compared with C‐dots in aqueous solution, the system exhibited superior efficacy in repairing corneal surface injury and increasing conjunctival goblet cell density [138]. This indicated that binary or multiple polymer blends are increasingly replacing single‐polymer systems to balance gelation strength, biocompatibility, and patient comfort.

Beyond poloxamers, alternative thermoresponsive polymers offer distinct advantages and limitations. Luo et al. employed gelatin‐g‐pNIPAAm (GN) thermosensitive to deliver epigallocatechin gallate (EGCG), achieving sustained drug release over 3 days, which was substantially longer than most P407‐based systems. However, PNIPAAm‐based polymers degrade slowly in ocular tissues, and their long‐term safety profile remains less established than that of poloxamers [139]. Similarly, Han et al. incorporated varying amounts of polyhedral oligomeric silsesquioxane (POSS) and PEG‐PPG copolymers, which were further loaded with FK506 micelles to form drug‐loaded hydrogels (MPEP‐FK506). The study revealed that the 2M‐MPEP (MPOSS content was 2 wt%) hydrogels required approximately 14 days for near‐complete degradation, representing one of the longest reported retention durations for thermoresponsive hydrogels. Furthermore, both in vitro and in vivo evaluations confirmed that the 2M‐FK506 hydrogel significantly elevated FK506 concentration in rabbit aqueous humor, accelerated restoration of corneal epithelial morphology, provided sustained anti‐inflammatory effects, and exhibited excellent biocompatibility with low toxicity [133].

pH‐responsive systems offer an alternative strategy, particularly for mitigating drug leakage during storage. Cellulose acetate phthalate (CAP), a derivative of cellulose ester, has been widely utilized as an enteric coating material for tablets and capsules due to its ability to dissolve in aqueous media at pH 6.0 or higher, thereby delaying drug release. This polymer can encapsulate hydrophobic drugs and protect them from environmental degradation. Kim et al. developed a pH‐responsive CLs for DED by printing a Cyclosporine A‐CAP (CsA‐CAP) solution onto the CLs surface, enabling controlled drug loading. Their study demonstrated that under storage conditions of pH 5.4 and 4 °C, CsA release was undetectable in solution over 24 h, with only 4.29% of the initial drug load released after 90 days. In contrast, under simulated physiological conditions (pH 7.4, 37 °C), approximately 94.0% of CsA was released within 24 h [136]. Likewise, Maulvi et al. employed Eudragit S100 NPs in CLs, demonstrating negligible drug release at pH 6.5 but sustained release for up to 14 days in rabbit tears [98].

While temperature‐ and pH‐sensitive in situ gelling systems often require suboptimal storage conditions (e.g., low temperatures or acidic environments) to maintain a liquid state, which may pose potential risks to ocular surface homeostasis, Ding et al. designed an innovative ion‐responsive system, which granted good stability of the drug and allowed long‐term storage in aqueous form without concerns of disintegration. They developed a TRPV1‐targeted ion‐responsive in situ gelling system by co‐assembly of functional peptide V1‐Cal, an inhibitor of TRPV1, and ion‐sensitive peptide called KFQ12. The system can form a supramolecular nanofiber scaffold, rapidly transform into a transparent hydrogel upon contact with ionic solutions and achieve sustained release of V1‐Cal over 7 days, which significantly alleviated inflammation and improved corneal epithelium recovery by blocking the TRPV1‐Ca2^+^‐Pyroptosis axis in DED [128].

#### 5.1.2 Targeted Anti‐Inflammatory Therapy via ROS‐Responsive Systems for DED

The overabundance of ROS in DED provides a precise trigger for targeted drug delivery. Mu et al. developed a ROS‐responsive MNs patch co‐loaded with CsA and EGCG for DED treatment, enabling invasive administration to the lacrimal gland area by penetrating the periocular skin for 600 µm (Figure 6D). In this system, EGCG serves dual functions as both an active therapeutic agent and a crosslinking component, forming ROS‐labile boronate ester bonds while providing anti‐inflammatory and antioxidant effects. Under elevated ROS conditions characteristic in Sjögren's syndrome‐related dry eye (SSDE), the boronate ester bonds underwent cleavage, triggering controlled release of both CsA and EGCG (Figure 6E). This ROS‐triggered mechanism enables sustained drug delivery to the lacrimal gland for nearly 72 h in SSDE mice compared with 120 h in normal mice, which indicated that CE‐MN could accelerate drug release in inflammatory environments (Figure 6F). Compared with conventional eye drops, the CE‐MN patch demonstrated superior efficacy in scavenging ROS, suppressing inflammation, and modulating the effector T cell/Treg balance, offering a comprehensive therapeutic strategy for SSDE (Figure 6G) [141]. In a separate approach, Cui et al. fabricated a novel ROS‐responsive PEG‐block‐PPS (PEG‐b‐PPS) micelles, which improved the solubility of celastrol (Cel) and enhanced intervention effect for DED. Furthermore, the study evaluated toxic effects on vital systemic organs, including the heart, liver, spleen, lung, and kidney, which confirmed the good biocompatibility [125].

The above studies revealed that significant progress has been made in the development of temperature‐, pH‐, and ion‐sensitive in situ gelling systems, which are designed to prolong drug residence time on the ocular surface, thereby enabling sustained release and improved bioavailability. Concurrently, ROS‐responsive materials, targeting oxidative stress and exert anti‐inflammatory effects, have also demonstrated significant therapeutic potential. These advances underscore the prospects of intelligent materials in managing DED. In the future, DDSs based on novel biomarker responsiveness remain to be explored. For instance, MMPs are significantly elevated in the tear fluid of DED patients and serve as key mediators of corneal epithelial damage and inflammation. Although no studies have yet formally reported the use of MMP‐responsive DDSs for DED, the specific overexpression of this enzyme positions it as a theoretically promising trigger for targeted drug release.

### 5.2 Infectious Keratitis

Infectious keratitis (IK) is a major cause of corneal blindness, responsible for an estimated 1.5–2.0 million new cases of monocular blindness worldwide each year [160, 161]. This condition arises from infection by pathogenic microorganisms such as bacteria, fungi, viruses, and amebae [162]. Bacteria such as *Pseudomonas aeruginosa* and *Staphylococcus aureus* can secrete virulence‐factor enzymes, including phospholipases, proteases (e.g., elastase, gelatinase, MMPs), and hyaluronidases, resulting in corneal stromal dissolution [163, 165]. And during bacterial infection, infected macrophages exhibit HIF‐1α‐mediated upregulation of glucose uptake and glycolysis. This metabolic shift leads to increased LDH expression, which converts pyruvate to lactic acid in the cytoplasm, ultimately acidifying the infection site to a pH as low as 5.8 [166, 168]. Studies also revealed that increased blood flow and inflammatory exudation in bacterial keratitis can raise local corneal temperature by approximately 0.8 °C [97]. Upon invasion of the corneal epithelium, pathogen‐associated molecular patterns (PAMPs) are recognized by host pattern recognition receptors (e.g., TLR4), activating the MyD88/NF‐κB and MAPK‐AP‐1 signaling pathways [24]. This leads to rapid upregulation of inflammatory cytokines including IL‐1β and TNF‐α, which in turn induce overexpression of MMPs such as MMP‐9 and MMP‐2 [22, 169]. Subsequent degradation of corneal stromal collagen fibers may result in severe complications including corneal perforation [170]. Concurrently, ROS generated by macrophages and neutrophils not only directly damage the corneal epithelium but also amplify oxidative stress by further stimulating inflammatory factors (TNF‐α, IL‐1β), leading to persistent tissue damage and delayed healing [171].

Currently, clinical management of IK primarily relies on pharmacological interventions that target pathogens, including broad‐spectrum antibiotics, antifungals, chlorhexidine, and ganciclovir [172, 173]. However, the corneal and tear film barrier significantly impede drug bioavailability and necessitate frequent administration to achieve therapeutic efficacy, which may reduce patient compliance and increase the risk of adverse effects [173, 174]. Moreover, the overuse of antibiotics accelerates microbial resistance, rendering current treatments unable to meet clinical needs [175, 177]. Furthermore, pathogen‐induced severe immune and inflammatory responses can exacerbate corneal damage, leading to stromal destruction and scarring [60, 178]. Hence, enhancing drug delivery and integrating multimodal therapies are crucial for IK management. MR‐DDSs, which can offer high biocompatibility and targeted release, present a promising strategy for IK. To date, several such systems have been investigated, including temperature‐sensitive hydrogels, pH‐responsive NPs, and ROS‐scavenging nanozymes.

#### 5.2.1 Enhancing Drug Residence via Thermo‐ and Ion‐Sensitive Systems for IK

The foremost challenge in treating IK with topical eye drops is the rapid precorneal clearance and low bioavailability, which leads to inadequate drug levels at the infection site and the need for frequent administration. Temperature‐ and ion‐sensitive in situ gelling systems provide a foundational solution to this problem. Poloxamers and CS/β‐glycerophosphate mixtures, which can form a transparent, mucoadhesive hydrogel on the ocular surface and extend the contact time of antibiotics from minutes to several hours, are widely studied for IK [179]. To optimize thermal and mechanical properties, Tavakoli et al. formulated a combined system of P407 and HPMC loaded with sertaconazole nanostructured lipid carriers (NLC) for fungal keratitis (FK) therapy [131]. This composite hydrogel exhibits a gelation temperature of 35.1 °C and rapid gel formation on the ocular surface, enabling sustained drug release for over 10 h and significantly extending drug residence time. In a large‐animal model study, Pereira et al. developed a voriconazole‐loaded thermosensitive gel based on PLGA‐PEG‐PLGA triblock copolymer. Following subconjunctival injection of 0.3 mL of 1.7% voriconazole gel (Vor‐Gel) in horses, voriconazole was released to the ocular tissues for at least 7 days, demonstrating its potential for sustained antifungal delivery [62].

Biopolymers like gellan gum and alginate gel instantly upon contact with tear film electrolytes, which allows for even faster gelation and prolonged retention, effectively increasing bioavailability and reducing dosing frequency [144]. For example, Victoria et al. developed an ion‐sensitive hydrogel for IK by combining deacylated gellan gum with κ‐carrageenan to encapsulate econazole. Upon contact with the tear fluid, the formulation undergoes ion‐triggered gelation, enabling sustained drug release. Positron emission tomography imaging in rats demonstrated that the ion‐sensitive hydrogel significantly prolonged ocular residence, with a mean half‐life of 50.16 ± 18.64 min and mean residence time of 72.37 ± 26.90 min, substantially exceeding the 20.73 ± 8.01 min and 29.9 ± 11.56 min observed with econazole aqueous solution [143].

#### 5.2.2 Applications of pH‐Responsive DDSs for IK

**FIGURE 7:**
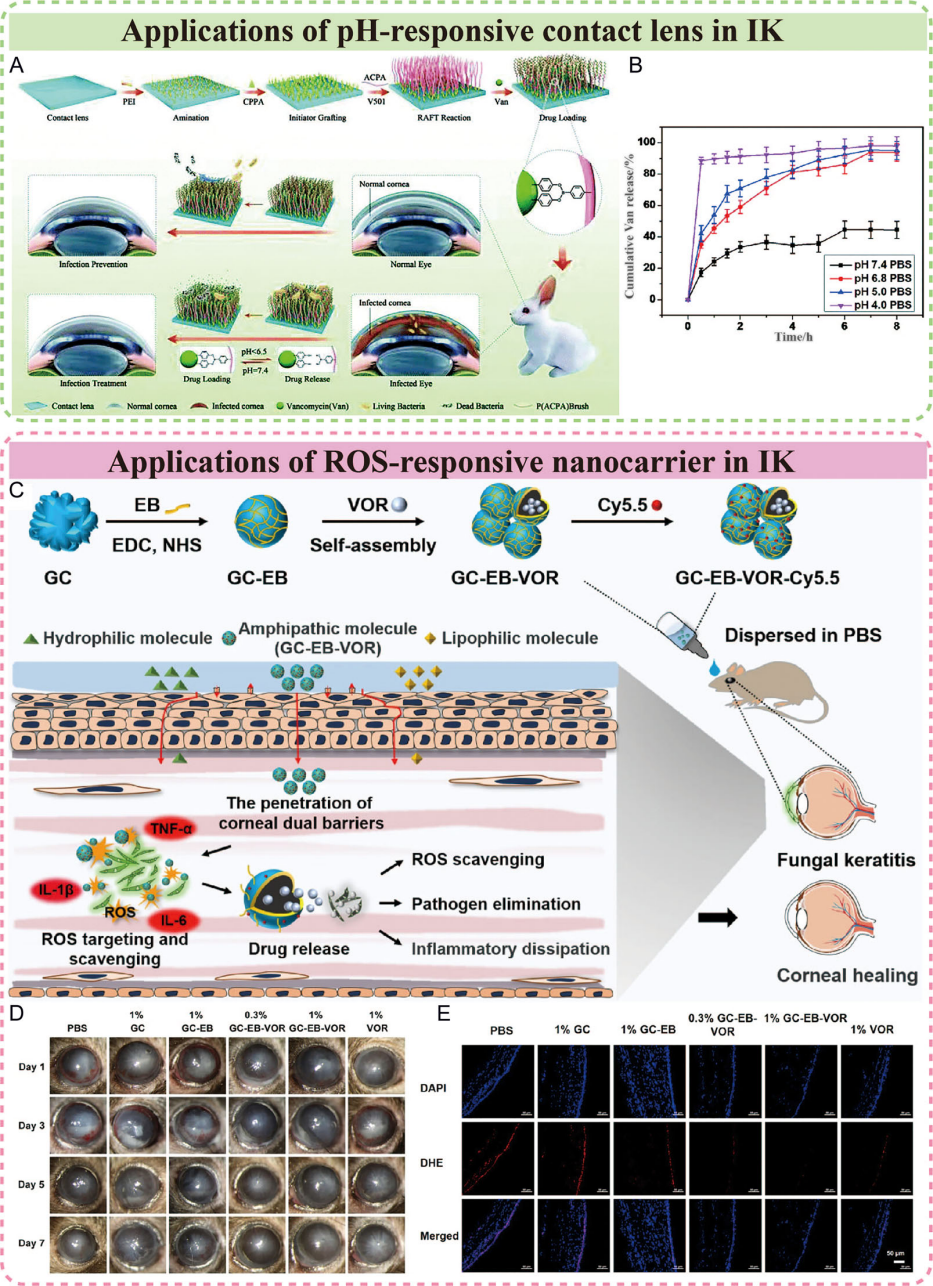
Applications of MR‐DDSs in IK. (A) Schematic illustration of preparation of pH‐responsive CLs. (B) Cumulative van release from PCVB‐CL in buffers with different pH values. Reproduced with permission from ref. [130] Copyright 2020, Royal Society of Chemistry. (C) Preparation and therapeutic mechanisms of GC‐EB‐VOR eye drops for FK treatment. (D) Time course of therapeutic efficacy in FK mice following different treatments. (E) Comparation of ROS levels in corneal tissue under different treatment conditions. The scale bar is 50 μm. Reproduced under the terms of the CC BY License 4.0, ref. [117] Copyright 2023, Springer Nature.

The reduced pH under infectious microenvironment provides an opportunity to design pH‐responsive DDSs, which can achieve precise and effective drug release. Interestingly, Siripongpreda et al. fabricated a dual‐purpose CLs integrated with cellulose nanofiber (CNF) /levofloxacin nanocomposites, enabling simultaneous diagnosis and treatment for IK. The lens was coated with a pH‐sensitive dye mixture containing 0.0125% methyl red, 0.0050% thymol blue, 0.0600% bromothymol blue, 0.1000% phenolphthalein, and 0.1% cetrimonium bromide, which produced visible color changes in response to pH variations. Under healthy conditions (pH ~ 7.4), the lens appeared bright green, while under infected conditions (acidic pH), they shifted to orange or red, providing a visual indicator for infection screening and severity monitoring. Drug release was also pH‐dependent, influenced by electrostatic interactions between LFX and CNF functional groups. At pH 5.0, the lens released 106.49 ± 0.77 μg of LFX over 24 h, significantly higher than the 70.53 ± 0.39 μg released at pH 8.0 [72]. Moreover, Guo et al. developed pH‐responsive CLs by integrating phenylboronic acid monomers and phenolic hydroxyl‐containing antibiotics. Through surface‐initiated reversible addition–fragmentation chain transfer polymerization (SI‐RAFT), a uniform brush layer of 4‐allylaminocarbonylphenylboronic acid (ACPA) was grafted onto the CLs surface. Vancomycin was subsequently conjugated to the ACPA brushes via dynamic boronic ester bonds formed between the phenylboronic acid groups and the phenolic hydroxyl moieties of the antibiotic, resulting in functionalized PCVB‐CLs (Figure 7A). The modified lenses exhibited pH‐dependent drug release characteristics. In PBS at pH 7.4, less than 50% of vancomycin was released over 8 h, whereas in pH 6.8, 5.0 and 4.0 PBS, over 80% of the drug was rapidly released within 1 h (Figure 7B). In a rat corneal infection model, the pH‐triggered vancomycin release significantly reduced inflammatory cell infiltration and mitigated local inflammatory responses [130].

#### 5.2.3 Targeted Anti‐Inflammatory Therapy via ROS‐Responsive DDSs for IK

The robust ROS during IK can serve as an endogenous trigger for ROS‐responsive DDSs. Nanocarriers constructed with thioketal or phenylboronic ester linkages respond to the elevated ROS and release antibiotics or anti‐inflammatory drugs. This strategy addresses both the infection and the detrimental host response simultaneously. For instance, Niu et al. designed a novel nanomedicine‐based eye drops (GC‐EB‐VOR), which employed glycol chitosan (GC) as a nanocarrier loaded with voriconazole (VOR), forming spherical NPs with an average diameter of approximately 38 nm (Figure 7C). The system not only efficiently penetrates both hydrophilic and lipophilic corneal barriers, but also achieves ROS triggered drug release, anti‐inflammatory and antioxidant functions by incorporating 4‐carboxyphenylboronic acid pinacol ester (EB)(105). It showed that GC‐EB‐VOR responded to elevated ROS levels by releasing VOR in a dose‐dependent manner and effectively reducing ROS in LPS‐activated HCECs. Compared with conventional VOR eye drops, the GC‐EB‐VOR eye drops showed enhanced corneal distribution and accumulation. Moreover, it significantly suppressed ROS, reduced levels of inflammatory cytokines (IL‐6, IL‐1β, and TNF‐α) and decreased immune cell infiltration in FK mice model (Figure 7D,E).

#### 5.2.4 Applications of Multistimuli‐Responsive DDSs for IK

Acanthamoeba keratitis (AK) is a severe corneal infection that can lead to blindness [73]. Conventional therapies such as 0.1% propamidine isethionate (PI) eye drops are limited by frequent drug administration, rapid tear clearance, and low bioavailability [180]. To address these challenges, Basant et al. developed an in situ gelling system based on a combination of thermosensitive F127 and pH‐sensitive CS. The researchers prepared and compared three PI‐loaded nanocarriers, including CS NPs (PI‐CS NPs), liposomes (PI‐LPs), and nanostructured lipid carriers (PI‐NLC) incorporated into the gel matrix. All three DDSs exhibited biphasic release profiles, with an initial relatively rapid release (11.7% to 25%) within the first 2 h, followed by a slower sustained release phase. The PI‐CS NPs gel demonstrated the highest cumulative drug release, exceeding 91% over 24 h, compared with approximately 40% for both PI‐LPs and PI‐NLC gels. In vitro anti‐amebic assays revealed that after 24 h of incubation, PI‐CS NPs achieved the highest inhibition rate of 92%, substantially outperforming other groups [63]. However,

In all, from strategies designed to prolong ocular surface residence time and enhance bioavailability, to systems responding to specific microenvironment and enabling precise drug release within the infectious site, substantial progress has been made in the research of MR‐DDSs for IK. In the future, efforts should focus on designing multi‐stimuli or sequential DDSs that enhance disease specificity and enable staged drug release that can better match the dynamic course of IK.

### 5.3 Corneal Neovascularization

Corneal neovascularization (CNV) is a pathological condition triggered by ocular disorders such as infection, CLs wear, or trauma [181]. The formation of new blood vessels compromises corneal transparency and alters its immune privilege, potentially leading to vision loss. Hypoxia, inflammation, and oxidative stress following corneal alkali burn and infections serve as key pathological drivers of CNV [182]. Studies have revealed that hypoxia activates hypoxia‐inducible factor (HIF), leading to upregulation of vascular endothelial growth factor (VEGF) and driving inflammatory, angiogenic, and fibrotic processes [183]. Concurrently, inflammation promotes the secretion of factors such as IL‐1β, IL‐6, CCL2, CCL3, CXCR2, MMP9, ICAM‐1, and CXCR4, which accelerate stromal degradation and CNV progression [184]. Extracellular matrix‐degrading enzymes including MMP‐9 and MMP‐13 can directly cleave type I collagen fibers, thereby creating physical space for nascent blood vessels to invade [185]. Additionally, excessive ROS generated after corneal alkali burns activate the transcription factor NF‐κB, which translocated to the nucleus and upregulates VEGF and MCP‐1, resulting in enhanced macrophage infiltration and CNV formation [66]. These microenvironmental features of CNV provide natural triggers for the design of MR‐DDSs [186]. Leveraging endogenous cues including elevated ROS and MMP levels to achieve spatiotemporally controlled drug release represents a key strategy to overcome the limitations of conventional eye drops, such as frequent dosing and poor corneal penetration.

#### 5.3.1 Enhancing Drug Residence via Thermo‐/pH‐ and Ion‐Sensitive Systems for CNV

To achieve more sustained drug delivery and prolonged ocular surface retention, thermos‐sensitive poloxamer is modified and combined with pH‐ and ion‐sensitive polymers like oxidized sodium alginate (OSA) and oxidized carboxymethyl chitosan (OCMC) [186, 187]. Anti‐VEGF agents such as bevacizumab (Avastin, Ava) can suppress CNV, but their efficacy is limited by poor corneal penetration and rapid clearance. To address these challenges, Xu et al. developed a thermosensitive hydrogel based on PEG‐PCL‐PEG (PECE) for sustained Ava delivery (Ava‐PECE). The formulation undergoes a sol–gel transition at physiological temperature (37 °C) and provides controlled release of Ava for up to 28 days in vitro, with release kinetics adjustable via PECE concentration. A single subconjunctival injection of Ava‐PECE maintained hydrogel integrity for over 3 weeks. Corneal Ava concentrations in the Ava‐PECE group were significantly higher than those in the Ava solution group at days 7 and 14 post‐administration. And on day 17, the Ava‐PECE hydrogel treatment resulted in a more pronounced reduction in neovascular area compared to Ava solution [145].

Furthermore, to improve drug penetration, Xu et al. incorporated ε‐polylysine (EPL), which was known for its membrane interaction and cellular uptake properties into a P407‐based thermosensitive gel (P‐EPL) for sustained release of bone morphogenetic protein 4 (BMP4). The P‐EPL gel exhibited a higher pore density than plain poloxamer gel and released over 70% of loaded bovine serum albumin (BSA) within 10 h in artificial tear fluid. In a CNV model, BMP4‐loaded P‐EPL gel more effectively inhibited neovascularization compared to BMP4 solution [146].

#### 5.3.2 Targeted Anti‐Inflammatory Therapy via ROS‐Responsive DDSs for CNV

**FIGURE 8:**
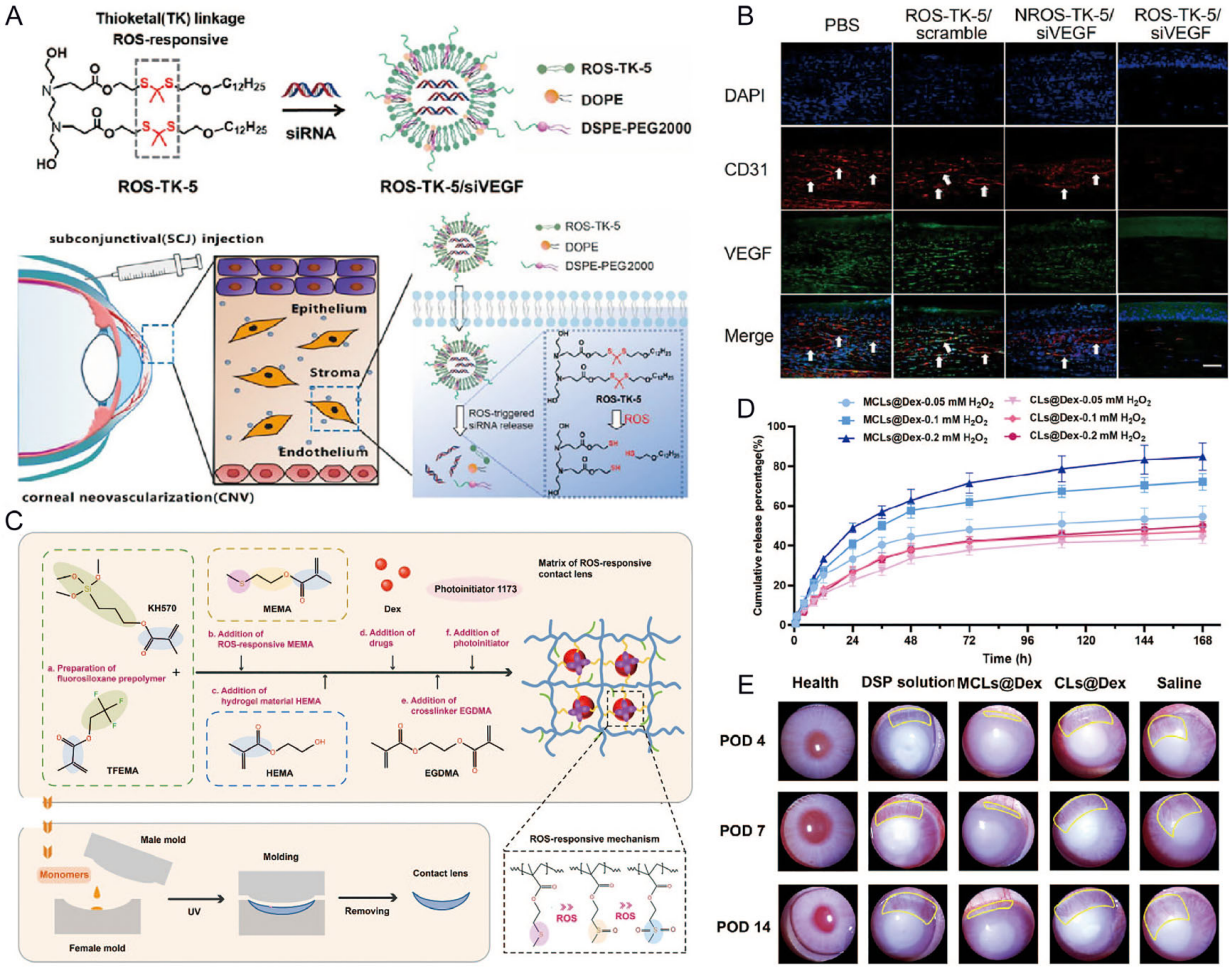
Application of ROS‐responsive DDSs for CNV. (A) Schematic illustration of ROS‐TK‐5/siVEGF NPs. (B) Comparation of VEGF expression levels in the corneal tissue after treatment on day 14. Scale bar: 100 μm. Reproduced with permission from ref. [149] Copyright 2022, American Chemical Society. (C) The preparation process and mechanism of ROS‐responsive CLs. (D) In vitro cumulative release percentage under different H_2_O_2_ concentrations. (E) the CNV development after modeling. Reproduced with permission from ref. [134] Copyright 2023, Elsevier.

The oxidative stress microenvironment in CNV makes it ideal for the design of ROS‐responsive DDSs. For instance, Liu et al. designed ROS‐responsive lipid NPs (ROS‐TK‐5/siRNA) that leverage the elevated ROS levels in CNV lesions to trigger siRNA release, enabling targeted silencing of VEGF expression (Figure 8A). The ROS‐TK‐5 lipid carrier not only protects siRNA from degradation, prolonging its gene silencing effect in the cornea, but also enables precise release, minimizing off‐target effects. When exposed to H_2_O_2_, approximately 50% of the encapsulated siRNA was released from the ROS‐TK‐5/siRNA NPs over 10 h. Furthermore, in alkali‐burn‐induced CNV mice model, the ROS‐TK‐5/siVEGF NPs were retained at the corneal lesion site for up to 48 h and significantly attenuated CNV (Figure 8B) [149].

In another study, Sun et al. developed a ROS‐responsive CLs for Dex delivery (MCLs@Dex) by incorporating the ROS‐sensitive monomer MEMA into the lens via in situ polymerization. In the high‐ROS microenvironment of inflammation, the hydrophobic thioether groups of MEMA are oxidized to hydrophilic sulfoxide/sulfone groups, triggering drug release (Figure 8C). This system enabled sustained Dex release over 168 h under 0.1 mM H_2_O_2_, achieving a cumulative release of 72.2%, compared with only 45.4% from nonresponsive CLs (CLs@Dex) (Figure 8D). In rabbit CNV models, the MCLs@Dex group achieved sustained drug levels above 100 ng/mL for 7 days, representing a 19.7‐fold longer ocular residence time and a 2.29‐fold higher bioavailability than Dex eye drops. Ultimately, MCLs@Dex significantly suppressed VEGF and MMP‐2/9 expression, reduced corneal ROS levels, and inhibited CNV area by 76.53% after 14 days of treatment, outperforming both non‐responsive lenses and eye drops (Figure 8E) [134].

#### 5.3.3 Applications of Multistimuli‐Responsive DDSs for CNV

To address the multifaceted pathological microenvironment of CNV, multistimuli‐responsive DDSs have emerged as a sophisticated therapeutic strategy. Fan et al. constructed a ROS‐responsive thermosensitive hydrogel DDS (Se‐PEG‐PPG, SePEP) for loading the anti‐angiogenic drug fenofibrate (Feno) to treat CNV. The hydrogel undergoes in situ gelation at 34.5 °C, prolonging drug retention on the ocular surface. Within this gel network, the selenoether groups not only interact with ocular mucins to enhance adhesion and sustain drug release but also scavenge ROS and accelerate drug release under high‐ROS conditions. Compared with ctrl (untreated CNV model) group, SePEP group, and PBS‐Feno group, the SePEP‐Feno group exhibited the smallest neovascularization area throughout the treatment period, which remained stable around 2 mm^2^ [74].

In conclusion, MR‐DDSs demonstrate promising applications in the treatment of CNV. The scope of these systems has expanded from conventional in situ gelling platforms to more sophisticated multistimuli‐responsive designs. Notably, gene therapy strategies are now being innovatively integrated with such smart DDSs, leveraging the pathological microenvironment to trigger the release of therapeutic siRNA, thereby significantly enhancing treatment precision and bioavailability. However, the design of responsive DDSs that target other critical pathological signals in CNV lesions, such as overexpressed MMPs, are still lacking. Moreover, the long‐term biocompatibility and toxicity of these novel DDSs have yet to be fully elucidated.

## 6 Translational Challenges and Regulatory Considerations

From a clinical translation perspective, temperature‐, pH‐, and ion‐responsive in situ gel‐forming systems offer several advantages, including good reproducibility, straightforward activation, and the ability to significantly prolong drug retention on the ocular surface while improving bioavailability. Additionally, these systems are stable, highly reproducible, and relatively easy to manufacture. Their main limitation, however, is weak disease specificity. In contrast, ROS‐ and enzyme‐responsive systems exhibit stronger disease relevance, as both ROS levels and enzyme activities are directly linked to inflammatory responses. Nonetheless, these systems face greater obstacles in clinical translation. ROS concentrations are transient and difficult to standardize, while enzyme activities vary considerably across the stage of diseases. Despite the remarkable progress of MR‐DDSs in preclinical settings, several critical challenges must be addressed before their clinical translation:

(1) Long‐term biosafety remains a paramount concern. Most studies report only short‐term efficacy (days to weeks) and safety data, leaving significant gaps in understanding long‐term degradation kinetics, byproduct toxicity, and cumulative effects of repeated dosing, particularly relevant for chronic DED management requiring daily administration for months or years. The long‐term immunometabolism and accumulation‐related toxicity of stimulus‐responsive polymers in vivo are not yet fully elucidated. Slow‐degrading polymers such as PCL can persist on the ocular surface for weeks, raising concerns for repeated dosing. For thermosensitive polymers such as pNIPAAm, although they offer excellent sol–gel transition behavior, their limited biodegradability means that residual polymer may accumulate after multiple instillations [83]. And PLGA‐based systems degrade into lactic and glycolic acid, which can lower local pH and trigger inflammatory responses upon accumulation [188].

(2) A key challenge for MR‐DDSs is achieving disease specificity, as many triggers such as temperature, pH and ROS, are common across multiple ocular diseases. However, the sustained‐release features of many systems help reduce systemic absorption. Furthermore, disease specificity can be enhanced by designing multistimuli‐responsive systems. Hierarchical or sequential DDSs that respond to multiple triggers in a specific order offer further advantages for complex diseases [189]. Once activated, they typically release the drug in a sustained manner allow for dosage adjustment according to disease progression [190].

(3) Batch‐to‐batch reproducibility remains a significant challenge. The synthesis of stimulus‐responsive DDSs typically involves multistep reactions that require precise control over molecular weight, grafting density, and the incorporation of responsive moieties, even minor variations in polymer composition can drastically alter gelation temperature, degradation kinetics, and drug release profiles. When scaling up from laboratory synthesis to industrial production, batch‐to‐batch variability becomes even more pronounced. Though using the same synthesis protocol, performance differences of up to 30% between batches can occur [191]. Sterilization and storage also present considerable challenges, particularly for thermosensitive and ROS‐responsive hydrogels. Autoclaving denatures thermosensitive polymers, while gamma irradiation tends to cause crosslinking or degradation of sensitive functional groups such as thioketal bonds. Moreover, issues such as drug stability during fabrication, burst release, drug loss during sterilization, and drug leaching during storage in packaging solutions have yet to be fully addressed [98].

(4) Regulatory barriers. With the rapid development of advanced materials, various drug carriers combined with the diversity of disease pathophysiological mechanisms has led to a vast array of stimulus–response DDSs combinations, which significantly increases the difficulty of manufacturing scale‐up, quality assessment, and batch‐to‐batch consistency control. Furthermore, the US Food and Drug Administration (FDA) has explicitly classified ophthalmic formulations intended for topical drug delivery, particularly those based on nanocarriers or stimulus‐responsive mechanisms, as complex products [192]. Compared with conventional formulations, such products face greater challenges in chemistry, manufacturing, and controls (CMC), including synthesis reproducibility, standardized characterization of critical quality attributes, sterilization compatibility and long‐term storage stability. These hurdles collectively raise the barrier for regulatory approval and impede the clinical translation.

(5) Another major gap is the lack of human clinical trials, as most evidence studies rely on rodent, rabbit or in‐vitro models that do not fully recapitulate human ocular anatomy, disease heterogeneity, and immune responses. Sakakura et al. provided quantitative comparisons between rabbit and human eyes including tear volume (5–10 vs. 7–30 μL), spontaneous blinking rate (4–5 vs. 6–15 times per hour), and corneal thickness (0.35–0.45 mm vs. 0.52–0.54 mm), which underscore the need for caution when interpreting preclinical findings [180]. Furthermore, the ratio of conjunctival to corneal surface area in rabbits is approximately twofold lower than in humans, which may affect tear distribution and drug absorption, and immune and inflammatory responses differ significantly between rabbits and humans [63, 181].

## 7 Conclusions and Future Perspectives

This review synthesized recent advances in the applications and mechanisms of microenvironment‐responsive materials for ocular drug delivery, providing clinicians, scientists, and materials researchers with a deeper understanding of these cutting‐edge developments. As advanced smart biomaterials, microenvironment‐responsive systems offer a transformative strategy to address the limitations of traditional ocular drug delivery. By leveraging pathological shifts in ocular microenvironmental, such as pH, temperature, and enzyme activity, these materials enable targeted, on‐demand, and sustained drug release, demonstrating considerable potential for managing a wide spectrum of ocular conditions. However, several critical challenges remain before their clinical translation, including the lack of disease specificity due to common triggers across multiple inflammatory conditions, insufficient long‐term safety data on degradation kinetics and byproduct toxicity, and the need for more robust manufacturing and regulatory standards. Furthermore, the majority of evidence remains preclinical, primarily from rodent or rabbit models, which poorly recapitulate human tear dynamics, blinking behavior, and immune responses. Rigorous clinical validation is urgently needed before these promising systems can reach patients.

In the future, more efforts should be paid to explore hierarchical or sequentially DDSs that respond to multiple pathological signals in a defined order, thereby achieving higher disease specificity and enabling staged release of combination therapeutics for synergistic outcomes. Additionally, current preclinical evaluation models, largely based on rodents and rabbits, poorly recapitulate human ocular physiology. The establishment of more physiologically relevant in vitro models using organoids and 3D bioprinting could bridge this gap, reducing reliance on animal models while better predicting clinical performance. Ultimately, a balanced approach that acknowledges both the transformative potential and the existing limitations of microenvironment‐responsive DDSs will be essential for guiding the field toward meaningful clinical translation.

## Author Contributions

J.L. drafted the manuscript and created all the figures. J.L., L.C., S.Z., and Y.F. discussed, edited, and supervised the manuscript. All authors read and approved the final manuscript.

## Funding

This research was funded by the National Natural Science Foundation of China (No. 82301166, 82471039 and 82271041), the Shanghai “Rising Stars of Medical Talents” Youth Development Program (SHWSRS (2025)‐71), the Clinical Research Special Program of Shanghai Health Commission (20254Y0006), the Shanghai Pujiang Program (25PJD070), the Biomaterials and Regenerative Medicine Institute Cooperative Research Project, and the Shanghai Jiao Tong University School of Medicine (2022LHA06).

## Conflicts of Interest

The authors declare no conflicts of interest.

## Data Availability

The data that support the findings of this study are available on request from the corresponding author. The data are not publicly available due to privacy or ethical restrictions.
